# Immunoglobulin M-degrading enzyme of *Streptococcus suis* (Ide*_Ssuis_*) impairs porcine B cell signaling

**DOI:** 10.3389/fimmu.2023.1122808

**Published:** 2023-02-16

**Authors:** Annika Katharina Breitfelder, Wieland Schrödl, Viktoria Rungelrath, Christoph Georg Baums, Gottfried Alber, Nicole Schütze, Uwe Müller

**Affiliations:** ^1^ Institute of Bacteriology and Mycology, Centre for Infectious Diseases, Faculty of Veterinary Medicine, University of Leipzig, Leipzig, Germany; ^2^ Institute of Immunology, Centre for Infectious Diseases, Faculty of Veterinary Medicine, University of Leipzig, Leipzig, Germany

**Keywords:** *Streptococcus suis*, Ide*_Ssuis_*, IgM, B cell receptor, receptor cleavage, B cell signalling

## Abstract

*Streptococcus suis* (*S. suis*) is an important porcine pathogen, causing severe disease like meningitis and septicemia primarily in piglets. Previous work showed that the IgM-degrading enzyme of *S. suis* (Ide*_Ssuis_*) specifically cleaves soluble porcine IgM and is involved in complement evasion. The objective of this study was to investigate Ide*_Ssuis_* cleavage of the IgM B cell receptor and subsequent changes in B cell receptor mediated signaling. Flow cytometry analysis revealed cleavage of the IgM B cell receptor by recombinant (r) Ide*_Ssuis_* _homologue as well as Ide*_Ssuis_* derived from culture supernatants of *S. suis* serotype 2 on porcine PBMCs and mandibular lymph node cells. Point-mutated rIde*_Ssuis_* _homologue_C195S did not cleave the IgM B cell receptor. After receptor cleavage by rIde*_Ssuis_* _homologue, it took at least 20 h for mandibular lymph node cells to restore the IgM B cell receptor to levels comparable to cells previously treated with rIde*_Ssuis_* _homologue_C195S. B cell receptor mediated signaling after specific stimulation *via* the F(ab’)_2_ portion was significantly inhibited by rIde*_Ssuis_* _homologue receptor cleavage in IgM^+^ B cells, but not in IgG^+^ B cells. Within IgM^+^ cells, CD21^+^ B2 cells and CD21^-^ B1-like cells were equally impaired in their signaling capacity upon rIde*_Ssuis_* _homologue B cell receptor cleavage. In comparison, intracellular B cell receptor independent stimulation with tyrosine phosphatase inhibitor pervanadate increased signaling in all investigated B cell types. In conclusion, this study demonstrates Ide*_Ssuis_* cleavage efficacy on the IgM B cell receptor and its consequences for B cell signaling.

## Introduction

*Streptococcus suis (S. suis)* is one of the most important porcine pathogens, causing mainly meningitis, arthritis, endocarditis, serositis, and septicemia, which lead to high economic losses (1). Disease mostly occurs in piglets around 4 to 10 weeks of age. Additionally, *S. suis* is a successful colonizer of mucosal surfaces in pigs of all ages, especially the nose and tonsils, but also the gastrointestinal and genital tract, without leading to clinical signs (2). *S. suis* is also an emerging zoonotic pathogen with outbreaks mostly in Asia (3). Currently, it is classified into 29 serotypes (*cps*), depending on the composition of its polysaccharide capsule, and various sequence types (4). The distribution of serotypes shows great geographical differences. Overall *S. suis* serotype 2 is the most important worldwide, causing human cases and most frequently isolated from diseased pigs (5). *S. suis* expresses a variety of virulence factors, e.g. the polysaccharide capsule (6–11).

*S. suis* also secretes a 124 kDa cysteine protease, designated IgM-degrading enzyme of *S. suis* (Ide*_Ssuis_*), with sequence homology to other streptococcal proteases such as IgG proteases IdeS of *S. pyogenes*, IdeE of *S. equi* subsp. *equi* and IdeZ of *S. equi* subsp. *zooepidemicus* (12–15). Ide*_Ssuis_* cleaves exclusively porcine IgM and is the only known bacterial IgM protease so far (12). Cysteine at position 195 in the active center is crucial as point mutation to serine (Ide*_Ssuis_* _C195S) leads to loss of cleavage activity (16). Cleavage occurs between constant domain C2 and C3 of IgM (17). IgM contains a C1q-binding motif in C3 to activate the classical complement pathway. Therefore cleavage by Ide*_Ssuis_* abrogates IgM labeling and complement activation on the bacterial surface (16, 17). The deletion mutant *S. suis* 10ΔIde*_Ssuis_* shows attenuated survival in porcine blood *in vitro* in the presence of increased IgM levels binding to the bacterial surface (16, 17). However, IgM cleavage by Ide*_Ssuis_* is not crucial for the virulence of *S. suis* serotype 2 in an intranasal infection model in piglets (16).

IgM is the most important natural antibody produced by B1 cells, and the first to be produced after antigen encounter by conventional B2 cells (18, 19). Levels of serum IgM binding to the surface of a *S. suis cps* 7 strain were significantly increased in 8-week-old in comparison to 4-week-old piglets (20). At that critical time of increased susceptibility to *S. suis* infection, specific maternal IgG antibody levels decline (21). Soluble pentameric IgM mediates aggregation and agglutination of antigen and activates the classical complement pathway (18, 22). Furthermore, the transmembrane monomeric IgM B cell receptor (BCR) is typically expressed on naïve mature porcine B cells before isotype switch occurs (23, 24). Upon antigen encounter, activation of B cells leads to somatic hypermutation, VDJ recombination and class switch to IgG, IgA or IgE or cells remain producing IgM (25–27). The IgM monomer as transmembrane protein represents the ligand-binding part of the IgM BCR. For signal transduction, the monomeric membrane-bound immunoglobulin requires association with a glycoprotein dimer of Igα (CD79α) and Igβ (CD79β) which each contain an immunoreceptor tyrosine-based activation motif (ITAM) (26). Receptor activation promotes recruitment of Src-family protein tyrosine kinases (PTK) such as Syk and Lyn which leads to phosphorylation of ITAMs (28). The next step in the signaling cascade is the formation of a membrane-associated signaling complex around B cell adaptor protein SH2-domain-containing leukocyte protein (SLP65, also known as BLNK). This signalosome contains signaling proteins such as phospholipase C-γ2 (PLC-γ2) (29). The following downstream phosphorylation cascade mediates second messenger pathways and eventually leads to B cell activation and differentiation (30). CD45 and CD19 function as positive coregulatory membrane proteins and enhance phosphorylation signals. Negative coregulator CD22 contains immunoreceptor tyrosine-based inhibitory motifs (ITIMs) which facilitate recruitment of protein tyrosine phosphatases (PTPs) like SHP1 and therefore inhibit B cell signaling (30). In contrast to BCR signaling after antigen encounter, tonic signaling occurs ligand-independent (29). It plays an important role in B cell survival and development (30). Treatment of B cells with the PTP inhibitor pervanadate increases tonic signaling (31).

This study was initiated by the finding that Ide*_Ssuis_* specifically cleaves soluble porcine IgM (12, 16, 17). Hence, we investigated cleavage activity of Ide*_Ssuis_* on porcine IgM^+^ B cells and tested the working hypothesis that cleavage of the IgM BCR interferes with IgM BCR-mediated signaling and thereby may modulate early adaptive host immunity in pigs infected with *S. suis*.

## Materials and methods

### Bacterial strains and growth conditions

*S. suis* serotype 2 strain 10 (wildtype, wt), kindly provided by Hilde Smith (Wageningen University and Research, Lelystad, Netherlands), was grown in Todd-Hewitt-Broth (THB) (Bacto, BD, Heidelberg, Germany, catalogue 249240) or on Columbia blood agar plates (Thermo Oxoid, Schwerte, Germany, catalogue PB5039A), at 37°C with 5% CO_2_. The isogenic in frame deletion mutant *S. suis* 10Δide*_Ssuis_* ∇ide*_Ssuis_* _C195S (∇ide*_Ssuis_* _C195S) was generated in a previous study (16) and cultivated the same way as the wildtype. *Escherichia coli* BL21 carrying either pETide*_Ssuis_* _homologue (12) or pETide*_Ssuis_* _homologue_C195S (16) were cultured in Luria-Bertani (LB) medium with 100 μg/mL ampicillin.

### Expression and purification of recombinant (r) proteins

Recombinant Ide*_Ssuis_* _homologue (rIde*_Ssuis_* _h) and recombinant Ide*_Ssuis_* _homologue_C195S (rIde*_Ssuis_* _h_C195S) were expressed in *Escherichia coli* BL21 pETide*_Ssuis_* _homologue and BL21 pETide*_Ssuis_* _homologue_C195S, respectively, after addition of isopropyl-β-D-thiogalactopyranoside (IPTG) during the exponential growth phase and lastly purified through Ni-affinity chromatography as described previously (12, 16).

### Concentration of wt Ide*_Ssuis_* from culture supernatant

*S. suis* 10 (wildtype, wt) and 10Δide*_Ssuis_* ∇ide*_Ssuis_* _C195S were grown in Iscove′s Modified Dulbecco′s Medium (IMDM, PAN Biotech, Aidenbach, Germany, catalogue P04-20150) supplemented with 5% heat-inactivated fetal calf serum (FCS) until the late exponential growth phase (OD_600nm_ 1.0 – 1.3) and then centrifuged at 3,400 x g for 10 min. The culture supernatant was collected and sterile filtered (0.45 µm, PES-Membrane, Sarstedt *via* Schubert Laborfachhandel, Leipzig, Germany, catalogue 1826-001). 24-fold concentration of Ide*_Ssuis_* or Ide*_Ssuis_* _C195S, respectively, was performed using Vivaspin 20^®^ filters with a 100 kDa cutoff (Sartorius, Göttingen, Germany, catalogue VS2042) according to manufacturer information. Activity of Ide*_Ssuis_* and Ide*_Ssuis_* _C195S in concentrated supernatants was verified by Western blot analysis.

### SDS-PAGE and Western blot analysis

Western blot analysis was conducted to detect Ide*_Ssuis_* or its derivatives as well as immunoglobulins and respective cleavage products. To verify cleavage activity of Ide*_Ssuis_* of *S. suis* on soluble IgM, 100 µl 24-fold concentrated culture supernatant was preincubated with 1:100 diluted porcine serum for 2 h at 37°C on a rotator. To detect secreted IgM in B cell culture supernatants, porcine mandibular lymph node cells and PBMCs were incubated with rIde*_Ssuis_* _homologue or rIde*_Ssuis_* _homologue_C195S for 45 min at 37°C, followed by removal of the recombinant proteins and cleavage products by extensive washing. Receptor cleavage was confirmed by flow cytometry as described below. Cells were resuspended in IMDM culture medium and incubated for 21 h. Cell culture supernatant was collected directly, 1 h and 21 h after washing and investigated in an anti-IgM Western blot.

Proteins were separated in SDS-Page under reducing conditions using 4% stacking gels and 10% separating gels, followed by semi-dry blotting onto a nitrocellulose membrane (Roth, Arlesheim, Switzerland, catalogue HP40.1). The membrane was blocked for 1 h at room temperature (RT) or overnight at 4°C under constant shaking in Tris-buffered saline with 0.05% Tween (TBST) supplemented with 5% skim milk powder. All primary and secondary antibodies and corresponding dilutions used are specified in **Supplementary Table 1**. Antibody incubations were performed in TBST with 1% skim milk powder at RT under constant shaking. Proteins were analysed using WesternBright™ chemiluminescence substrate (Biozym, Hessisch Oldendorf, Germany, catalogue 541014) according to manufacturer’s instructions. Detection was carried out using Fusion SL imaging system and FusionCapt Advance Solo 4 software (both by Vilber Lourmat, Eberhardzell, Germany). Marker bands were overlaid automatically.

### Purification of immunoglobulins

All chromatographic purification procedures were carried out with the ÄCTA prime plus chromatography system (GE Healthcare, Little Chalfont, United Kingdom). Size exclusion chromatography (SEC) was performed with the HiLoad 16/600 Superdex 200pg column (GE Healthcare *via* VWR, Darmstadt, Germany, catalogue 28-9893-35) under the following conditions: flow rate 1 mL/min, 2 mL sample load, collected eluate 2 mL/tube and phosphate buffered saline (PBS, pH 7.35) as running buffer. For affinity chromatography and pre-absorption steps, proteins were covalently bound to CNBr-activated Sepharose 4B (GE Healthcare, Little Chalfont, United Kingdom, catalogue 17-0430-01) according to manufacturer instructions (instruction #71-7086-00 AF). Elution was performed with 0.1 M citric acid/sodium phosphate, pH 2.5 and the eluates were neutralized with 0.5 M trisodium phosphate.

For purification of IgM, porcine serum was precipitated with 6% polyethylenglycole 4000 (PEG4000, Carl Roth, Arlesheim, Switzerland, catalogue 0156), the precipitate was then solubilized in PBS and purified by affinity chromatography with Sepharose columns covalently coupled to Protein G (HiTrap protein G column, GE Healthcare *via* VWR, Darmstadt, Germany, catalogue 29–0485-81), followed by size exclusion chromatography. The eluates with corresponding size to IgM were pooled and concentrated with 10 kDa cutoff centrifugal filters (VIVASPIN 20^®^, Sartorius, Göttingen, Germany, catalogue VS2001).

Anti-porcine IgM F(ab')_2_ polyclonal antibodies were generated as described in (16). Briefly, purified porcine IgM was cleaved by rIde*_Ssuis_* (20 µg rIde*_Ssuis_*/mg IgM) and thereby generated IgM F(ab')_2_ fragments were purified by SEC (see above) and covalently coupled to CNBr-activated Sepharose 4B columns. Within a previous study (16), the purified IgM F(ab')_2_ fragments were also used as antigen for rabbit immunization consisting of one priming and two booster immunizations. A part of the generated rabbit antiserum was precipitated with 40% saturated ammonium sulfate solution and the precipitate was solubilized in 0.1 M sodium acetate buffer, pH 4.5. One milligram pepsin (3829 U/mg, Fluka Analytical *via* Sigma-Aldrich, Darmstadt, Germany, catalogue 77152) was added to 50 mg gammaglobulin, followed by incubation for 24h at 37°C. Resulting rabbit anti-IgM F(ab')_2_ antibodies were first purified by affinity chromatography with IgM-F(ab')_2_-coupled Sepharose 4B columns (see above). After neutralization (pH 7.5), the eluate was preabsorbed with porcine IgG-coupled Sepharose 4B columns (10 mg IgG/mL Sepharose) and the flow through was concentrated with 10 kDa cutoff centrifugal filters to 2 mL (VIVASPIN 20^®^, Sartorius, Göttingen, Germany, catalogue VS2001). After a second purification step *via* SEC, a part of the rabbit anti-IgM F(ab')_2_ antibody preparation was conjugated wit NHS-fluorescein (ThermoFisher Scientific, Schwerte, Germany, catalogue 46410) and purified following manufacturer instructions. The degree of labeling was calculated from spectrophotometric measurements as specified in the manufacturer’s instructions. The other part of the rabbit anti-IgM F(ab')_2_ antibody preparation remained pure and was used in the stimulation experiments. The final purified polyclonal rabbit anti-porcine IgM F(ab')_2_ antibody preparation (following referred to as anti-IgM F(ab')_2_) was stored at 4°C in PBS supplemented with 0.1% ProClin™.

### Cells

Mandibular lymph nodes and whole blood was collected from commercially farmed pigs (German Landrace x Pietrain or German saddleback pig) 11 months of age (data shown in **Figures 1**, **2**, and **Supplementary Figures 3, 6A**) or between 5 and 16 weeks of age (data shown in **Figures 3**-**5**, and **Supplementary Figures 2, 6B, 7–10**).

**Figure 1:**
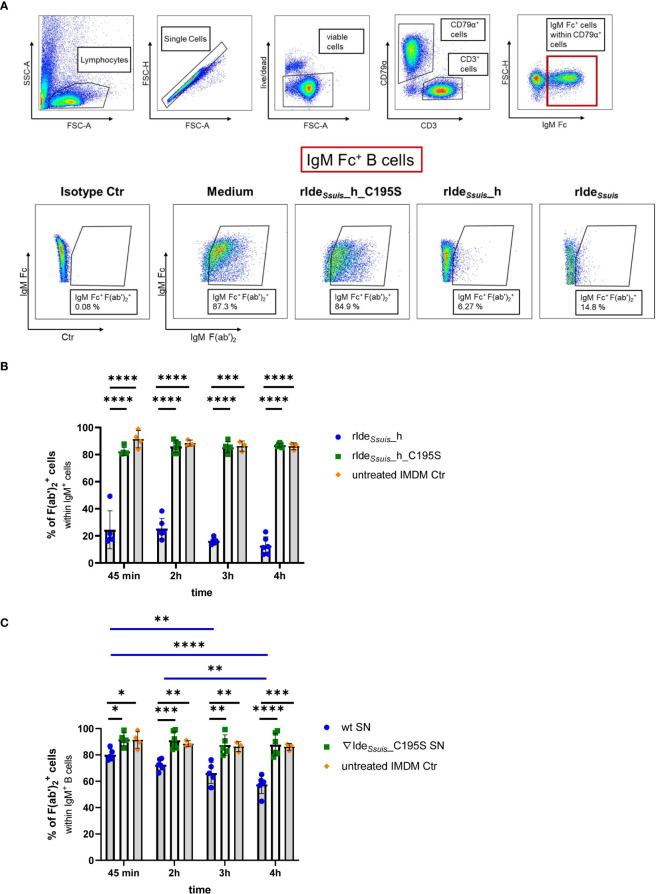
Recombinant and *S. suis* derived Ide*_Ssuis_* cleave the B cell receptor on porcine CD79α^+^IgM^+^ B cells. **(A)** Gating strategy to analyze viable IgM^+^ B cells (IgM^+^CD79α^+^CD3^-^) after treatment with rIde*_Ssuis_* _homologue (rIde*_Ssuis_* _h), rIde*_Ssuis_* or non-functional point-mutated rIde*_Ssuis_* _homologue_C195S (rIde*_Ssuis_* _h_C195S). Representative pseudocolor plots of mLN cells of 11-month-old pigs are shown. 1 x 10^6^ cells were incubated with 4 µg of each protein for 45 min at 37 °C. Viable cells were gated for the B cell marker CD79α and then for porcine IgM Fc. To detect cleavage, anti-IgM F(ab’)_2_ was used (lower panels, see Material and Methods). IgM^+^ B cells were gated for an intact (IgM Fc^+^ F(ab')_2_^+^) BCR. After receptor cleavage, cells lose the signal for IgM F(ab')_2_. **(B)** BCR cleavage with recombinant proteins. Significant reduction of IgM F(ab')_2_^+^ cells by rIde*_Ssuis_* _h in contrast to rIde*_Ssuis_* _h_C195S on porcine B cells (gating as described in 1A) is detectable after 45 min and persist until 4 h. Mandibular lymph node cells of 11-month-old pigs (n = 6) were incubated with 4 µg/1x10^6^ cells of rIde*_Ssuis_* _h or rIde*_Ssuis_* _h_C195S for 45 min, 2 h, 3 h, or 4 h. Cells were analyzed for the percentage of IgM F(ab')_2_^+^ cells within IgM Fc^+^ B cells using flow cytometry. Statistical analysis was conducted with mixed ANOVA with Turkey`s multiple comparisons test. Bars and error bars represent mean and standard deviation, significant differences are indicated (p< 0.001 ***, p < 0.0001 ****). **(C)** BCR cleavage with culture supernatant (SN). Significant reduction of IgM F(ab')_2_^+^ cells by *S. suis* 10 (wt) supernatant in contrast to *S. suis* 10Δide*_Ssuis_* ∇ide*_Ssuis_* _C195S (10∇ide*_Ssuis_* _C195S) supernatant on porcine B cells (gating as described in 1A) is detectable after incubation for 45 min and increases until an incubation period of 4 h. Mandibular lymph node cells of 11-month-old pigs (n = 6) were incubated with 100µl/1x10^6^ cells of 24-fold concentrated culture supernatants for 45 min, 2 h, 3 h, or 4 h. Cells were analyzed for the percentage of IgM Fc^+^ F(ab')_2_^+^ cells within IgM^+^ B cells using flow cytometry. Statistical analysis was conducted with mixed ANOVA with Turkey`s multiple comparisons test. Bars and error bars represent mean and standard deviation, significant differences are indicated (p< 0.1 *, p < 0.01 **, p < 0.001 ***, p < 0.0001 ****). Results of statistical analyses considered not significant are not shown (p > 0.05).

**Figure 2:**
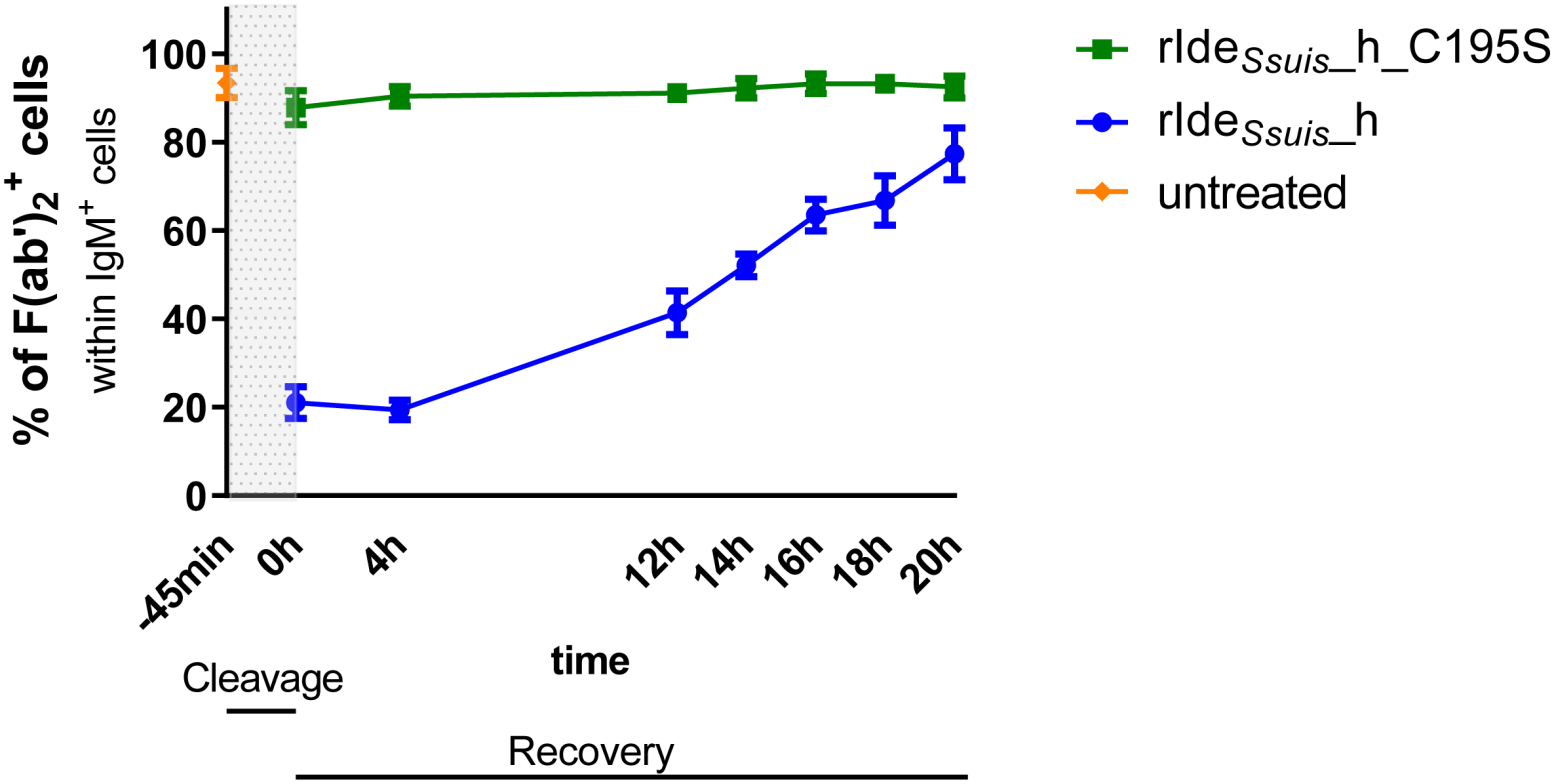
Recovery of IgM B cell receptor expression on porcine lymph node cells takes approximately 20 hours after rIde*_Ssuis_* _homologue mediated cleavage. Porcine mandibular lymph node cells of 11-month-old pigs (n = 5 for time points -45 min, 0 h, 4 h, 20 h; n = 3 for time points 12 h, 14 h, 16 h, 18 h) were incubated for 45 min with 4 µg/1x10^6^ cells rIde*_Ssuis_* _homologue (rIde*_Ssuis_* _h) or non-functional rIde*_Ssuis_* _homologue_C195S (rIde*_Ssuis_* _h_C195S). After washing, cells were incubated for the given times in culture medium. Cells were stained and analyzed as described in **Figure 1A**.

For the preparation of peripheral blood mononuclear cells (PBMC) the blood was centrifuged at 400 x g for 10 min. Plasma was removed and the blood was diluted with the same amount of PBS. Density gradient centrifugation was performed over Ficoll (PAN Biotech, Aidenbach, Germany, catalogue P04-601000) at 900 x g for 35 min, followed by gently collecting the interphase with a pipette. Cells were washed with IMDM + 10% heat-inactivated FCS (500 x g, 12 min, RT) and again with PBS (400 x g, 10 min, RT). If necessary, erythrocyte lysis was performed by resuspending the cells in 1-2 mL lysis buffer (PBS supplemented with ammonium chloride, potassium hydrogen carbonate and ethylenediaminetetraacetic acid (EDTA)) and incubating for 2-3 min. Lysis was stopped by adding 1 mL heat-inactivated FCS and filling up with 10 mL IMDM + 10% FCS. After two washing steps (400 x g, 6 min, RT), cells were used directly or resuspended in heat-inactivated FCS supplemented with 10% dimethyl sulfoxide (DMSO, Sigma, Darmstadt, Germany, catalogue D2650-100ML) (freezing medium) and frozen at - 80°C.

Lymph nodes were minced and transferred into digestion medium consisting of IMDM supplemented with 1% penicillin/streptomycin, 50 µM gentamicin and DNase (final concentration 111.1 U/mL). After incubation for 15 min at 37°C, samples were homogenized using gentleMACS™ Dissociator according to manufacturer information. This step was repeated, followed by filtration through sterilized cotton wool and rinsing with PBS. Cells were washed three times with PBS at 400 x g and 4°C for 12 min and then resuspended in freezing medium and stored at -80°C.

### BCR cleavage

Cells were thawed, counted with Trypan Blue and adjusted to 1 x 10^7^ cells/mL in IMDM medium supplemented with 10% heat-inactivated FCS and 1% penicillin-streptomycin (PAN Biotech, Aidenbach, Germany, catalogue P06-07100) (IMDM^++^). Additionally, 10 ng/mL recombinant porcine IL-2 (R&D Systems, Minneapolis, US, catalogue 652-P2) was added to increase cell viability. Four million cells were incubated with 16 µg rIde*_Ssuis_* _homologue, 16 µg rIde*_Ssuis_* _homologue_C195S or 100 µl 24-fold concentrated culture supernatant from *S. suis* wt or 10Δide*_Ssui_*_s_∇ide*_Ssuis_* _C195S, respectively, for 45 min, 2 h, 3 h, or 4 h at 37°C with 5% CO_2_. Then, cells were transferred into a 96 well plate with 4x10^5^ cells/well on ice, washed two times with cold PBS and stained 20 min at 4°C in 100 µl 1:500 prediluted viability dye. Cells were washed one time with cold PBS supplemented with 3% heat-inactivated FCS and 0.1% sodium acid (FACS buffer) and two times with cold PBS, followed by fixing for 30 min at 4°C with 100 µl 2% paraformaldehyde (PFA) and three times washing with cold FACS buffer.

Next, cells were stained for 15 min at 4°C in 20 µl of staining mix 1 containing anti-IgM Fc and anti-IgM F(ab')_2_, F(ab')_2_ control mix containing anti-IgM Fc and anti-rabbit IgG isotype control or IgM Fc control mix containing mouse IgG1 isotype control and anti-IgM F(ab')_2_, respectively, diluted in FACS buffer. After two washing steps in FACS buffer, cells were blocked for 10 min at 4°C in 20 µl FACS buffer containing 30% porcine heat-inactivated serum (Fc block I). The cells were then stained in 20 µl of staining mix 2 containing anti-mouse IgG1 and anti-CD3 or CD3 control mix containing anti-mouse IgG1 and mouse IgG2a isotype control, respectively, diluted in Fc block I. Following 15 min incubation at 4°C, cells were washed once with FACS buffer and twice with FACS buffer containing 0.5% saponin (saponin buffer) to prepare for intracellular staining. After blocking with saponin buffer containing 30% porcine heat-inactivated serum (Fc block II) for 5 min at 4°C, cells were stained for 30 min at 4°C in 20 µl of staining mix 3 containing anti-CD79α diluted in Fc block II or CD79α fluorescence minus one (FMO) control mix without anti-CD79α. After washing once with saponin buffer and once with FACS buffer, cells were resuspended in 100 µl FACS buffer and measured by flow cytometry (LSRFortessa, BD Biosciences, Heidelberg, Germany). Analyses were carried out using FlowJo™ 10 software (BD Biosciences, Heidelberg, Germany) and gates were placed based on specific isotype controls. First, lymphocytes were gated forward scatter area (FSC-A) versus side scatter area (SSC-A) based on size and granularity. Next, single cells were gated forward scatter height (FSC-H) versus forward scatter area (FSC-A) to exclude doublets. Viable cells were distinguished using CD79α as B cell marker and CD3 as T cell marker in B cells (CD79α^+^CD3^-^) and T cells (CD79α^-^CD3^+^). IgM Fc^+^ CD79α^+^ B cells were analyzed for an intact (IgM Fc^+^ F(ab')_2_^+^) or cleaved (IgM Fc^+^ F(ab')_2_^−^) IgM B cell receptor.

For investigation of receptor cleavage on CD21^+^ or CD21^-^ cells, staining mix 2 contained anti-mouse IgG1 and anti-CD21 in Fc block I and staining mix 3 contained Streptavidin in Fc block I. All other steps were performed as described above.

All antibodies used are specified in **Supplementary Table 2**. All washing steps were performed for 4 min at 400 x g or 500 x g after fixation, respectively.

### B cell receptor recovery

Cells were thawed, counted and adjusted to 1 x 10^7^ cells/mL in IMDM^++^ + poIL2 as described above. Four million cells were incubated with 16 µg rIde*_Ssuis_* _homologue or rIde*_Ssuis_* _homologue_C195S, respectively, for 45 min at 37°C with 5% CO_2_. After removal of the recombinant proteins by two washing steps with IMDM^++^, cells were incubated in IMDM^++^ supplemented with porcine IL-2 to promote cell survival for up to 20 hours. For each investigated time point, 4 x 10^5^ cells/well were transferred into a 96 well plate onto ice and washed two times with cold PBS. All following steps were performed analogous to BCR cleavage experiments.

### Incubation of myeloid cells and B cells with soluble IgM

PBMC were thawed, counted and adjusted to 1 x 10^7^ cells/mL in IMDM^++^ + poIL2 as described above. Four million cells were incubated with 0.5 mg/mL IgM for 30 min at 37°C or remained untreated, respectively. After washing with IMDM^++^, cells were transferred into a 96 well plate with 4 x 10^5^ cells/well and washed two times with cold PBS. Staining for viability and fixating with 2% PFA were performed as described above. Next, cells were stained for 15 min at 4°C in 20 µl of staining mix 1 containing anti-IgM Fc or IgM Fc control mix containing mouse IgG1 isotype control, respectively, diluted in FACS buffer. After two washing steps in FACS buffer, cells were blocked for 10 min at 4°C in 20 µl FACS buffer containing 30% porcine heat-inactivated serum (Fc block I). The cells were then stained in 20 µl of staining mix 2 containing anti-mouse IgG1 and anti-CD172a or CD172a control mix containing anti-mouse IgG1 and mouse IgG2b isotype control, respectively, diluted in Fc block I. Following 15 min incubation at 4°C, cells were washed once with FACS buffer and twice with saponin buffer. After blocking with saponin buffer containing 30% porcine heat-inactivated serum (Fc block II) for 5 min at 4°C, cells were stained for 30 min at 4°C in 20 µl of intracellular staining mix containing anti-CD79α diluted in Fc block II or CD79α FMO mix without anti-CD79α. All antibodies used are specified in **Supplementary Table 2**. Cells were measured by flow cytometry as described above.

### B cell stimulation

We investigated the impact of BCR cleavage by rIde*_Ssuis_* on the stimulatory capacity of anti-IgM F(ab')_2_ and pervanadate. PBMC were used freshly isolated or thawed and adjusted to 1 x 10^7^ cells/mL in IMDM^++^ + poIL2 as described above. Four million cells were incubated with 16 µg rIde*_Ssuis_* _homologue or rIde*_Ssuis_* _homologue_C195S, respectively, for 45 min at 37°C with 5% CO_2_. After two washing steps with cold PBS, cells were stained for 20 min at 4°C in prediluted viability dye with anti-IgM Fc or mouse IgG1 isotype control, respectively. Then, cells were washed two times with cold IMDM^++^ medium and resuspended in IMDM^++^ + poIL-2. Cells were seeded into a 96 well plate with a concentration of 4 x 10^5^ cells/well and incubated for 20 min at 37°C.

To prepare pervanadate, 10 µl 0.1 M sodium orthovanadate (Sigma-Aldrich, Darmstadt, Germany, catalogue 450243-10G) was incubated with the same amount of 1:50 prediluted (0.6% final conc.) hydrogen peroxide (Carl Roth, Arlesheim, Switzerland, catalogue 8070.3) for 15 min at RT, followed by inactivation of surplus H_2_O_2_ by adding 30 µl catalase (Sigma-Aldrich, Darmstadt, Germany, catalogue C9322-1G) (20 mg/mL catalase in KH_2_PO_4_ buffer). The solution was centrifuged at 5000 x g for 5 min and the supernatant diluted 1:20 in buffer A (20 mM HEPES-NaOH, 120 mM NaCl, pH 7.4).

Cells were stimulated with anti-IgM F(ab')_2_ (10 µg/mL final concentration) for 1, 2, or 4 min, respectively. Stimulation with 10 µl pervanadate (0.1 mM final concentration) was performed for 5, 10, 15, or 20 min. All agents used for stimulation are specified in **Supplementary Table 3**. Stimulation was stopped all together by adding the same amount 4% PFA and incubation for 10 min at RT, followed by two washing steps with cold PBS and fixation with 2% PFA for 30 min at 4°C. After two times washing with cold FACS buffer, cells were stained for 15 min at 4°C in 20 µl of staining mix 1 containing anti-porcine IgG or the specific isotype control, diluted in FACS buffer. Next, cells were washed two times with cold FACS buffer, followed by 10 min incubation at 4°C in 20 µl Fc block I. Cells were then stained for 15 min at 4°C in 20 µl of staining mix 2 containing anti-mouse IgG1 and streptavidine, diluted in Fc block I. After two washing steps with cold PBS, cells were permeabilized with 100 µl ice-cold 99% methanol for 30 min on ice, followed by centrifugation, removal of supernatant and two times washing with cold FACS buffer. Next, cells were incubated in 20 µl FACS buffer with 30% murine serum (Fc block III) for 10 min at 4°C and then stained for 30 min at RT in 20 µl staining mix 3 containing anti-phospholipase C-γ2 or mouse IgG1 isotype control, respectively, diluted in Fc block III. After washing with FACS buffer twice, cells were incubated for 15 min at 4°C in 20 µl of staining mix 4 containing anti-CD21 or the respective isotype control, diluted in Fc block I. Cells were measured by flow cytometry as described above.

Cells for rIde*_Ssuis_* cleavage control as well as IgM Fc and F(ab')_2_ isotype controls were stained as described for BCR cleavage. All antibodies used for staining are specified in **Supplementary Table 2**. All washing steps were performed for 4 min at 400 x g or 500 x g after fixation, respectively.

### Statistical analysis

All data represent at least three independent experiments. All statistical analyses were performed with GraphPad Prism 9 (Dotmatics, Boston, Massachusetts, US, www.graphpad.com). Distribution of data was tested both with Shapiro-Wilk and Kolmogorov-Smirnov test. If data achieve normal distribution, unpaired two-tailed t-test was applied. In case of no normal distribution, Mann-Whitney test was conducted. For comparison of more than two measurements, mixed analysis of variance (ANOVA) with subsequent Turkey´s multiple comparisons test was used. Figures show mean and standard deviation or median. Probabilities below 0.05 were considered significant (p**<** 0.05 *, p**<** 0.01 **, p**<** 0.001 ***, p**<** 0.0001 ****). Flow cytometric data was analyzed with FlowJo™ 10 software (BD Biosciences, Heidelberg, Germany, www.flowjo.com).

## Results

### Recombinant and *S. suis* derived Ide*_Ssuis_* cleaves the porcine IgM B cell receptor

Ide*_Ssuis_* cleaves soluble porcine IgM and leads to a reduction of porcine IgM bound to the bacterial surface (12, 16). The cysteine protease IdeS of *Streptococcus pyogenes* is known to cleave soluble IgG as well as the IgG B cell receptor (32). Thus, we hypothesized that Ide*_Ssuis_* is also able to cleave porcine IgM as a transmembrane protein within the B cell receptor complex. This hypothesis was first investigated with the following recombinant derivatives of Ide*_Ssuis_*: the intact IgM protease domain (rIde*_Ssuis_* _homologue), the point-mutated, inactive IgM protease domain (rIde*_Ssuis_* _homologue_C195S) or the intact IgM protease domain together with the large C-terminus of still unknown function (rIde*_Ssuis_*) (**Supplementary Figure 1**). Specifically, porcine mandibular lymph node cells and PBMCs were treated with these recombinant proteins and stained for IgM Fc^+^ B cells. Representative plots of flow cytometry analyses of mandibular lymph node cells are shown in **Figure 1A**. Viable lymphocytes were distinguished in B cells (CD79α^+^CD3^-^) and T cells (CD79α^-^CD3^+^). As Ide*_Ssuis_* cleaves IgM between constant domain C2 and C3 (16), we chose a monoclonal anti-porcine IgM Fc antibody (K52 1C3), putatively recognizing the membrane bound C3-C4 IgM fragment after Ide*_Ssuis_* treatment, to detect IgM^+^ B cells. Polyclonal rabbit anti-IgM F(ab')_2_- specific antibodies were used to detect the F(ab) part of IgM and therefore an intact IgM BCR (IgM Fc^+^ F(ab')_2_^+^). Lower panels of **Figure 1A** show representative pseudocolor plots of IgM^+^ B cells after treatment with the indicated recombinant proteins. We verified the cleavage activity of rIde*_Ssuis_* containing the large C-Terminus in a representative number of experiments and found no significant difference in IgM Fc^+^ F(ab')_2_^+^ B cells in comparison to the cleavage activity of rIde*_Ssuis_* _homologue (data not shown). Therefore, we continued the following experiments only using rIde*_Ssuis_* _homologue.

Indeed, flow cytometry analysis revealed a significant reduction of approximately sixty percent in IgM Fc^+^ F(ab')_2_^+^ cells after treatment with rIde*_Ssuis_* _homologue for 45 min, whereas non-functional rIde*_Ssuis_* _homologue_C195S had no significant effect on the number of IgM Fc^+^ F(ab')_2_^+^ cells (**Figure 1B**). After continuous incubation with rIde*_Ssuis_* _homologue for 4 h, the reduction in IgM Fc^+^ F(ab')_2_^+^ cells increased to over seventy percent. These findings for the 45 min incubation time period could be reproduced in PBMCs as well as in mLN cells of piglets of a different age (**Supplementary Figure 2**).

Taken together, these data indicate that rIde*_Ssuis_* _homologue as well as rIde*_Ssuis_* are able to cleave membrane-bound IgM and that cysteine at position 195 is crucial for this cleavage activity, analogous to cleavage of soluble IgM (16). The large C-terminal domain of Ide*_Ssuis_* is dispensable for this cleavage activity on the IgM BCR. Both active recombinant variants of Ide*_Ssuis_* efficiently remove the F(ab')_2_ part of the IgM BCR. The Fc part remained unaffected and was still detectable on B cells after up to 4 h treatment with rIde*_Ssuis_* _homologue (**Supplementary Figure 3**).

We further assessed whether Ide*_Ssuis_* secreted by *S. suis* is able to cleave the IgM BCR. *S. suis cps*2 strain 10 (wt) and the isogenic *S. suis* mutant 10Δide_Ssuis_∇ide_Ssuis__C195S, which expresses a point-mutated, inactive full-length variant of Ide*_Ssuis_* (16), were grown in IMDM medium. The supernatant was 24-fold concentrated via 100 kDa cutoff centrifugal filters to retain Ide_Ssuis_ or Ide_Ssuis__C195S (both 124 kDa) while eliminating the largest possible proportion of other secreted bacterial components, especially potential B cell damaging factors like suilysin. Cleavage of soluble IgM by those culture supernatants was confirmed in anti-IgM Western blot analyses under reducing conditions (**Supplementary Figure 4**).

Concentrated bacterial culture supernatants were incubated for 4 h with mandibular lymph node cells. Anti-Ide*_Ssuis_* Western blot analysis revealed characteristic Ide_Ssuis_ protein bands between 175 and 270 kDa as described by Seele et al. (**Supplementary Figure 5**) (12), which suggests that Ide_Ssuis_ and Ide_Ssuis__C195S are fairly stable under these conditions. For comparison, recombinant homologue proteins were also incubated with mandibular lymph node cells and examined in parallel. Typical protein bands were detectable between 52 and 66 kDa as described by Rungelrath et al. (16).

Analogous to BCR cleavage with recombinant enzymes, mandibular lymph node cells were incubated with concentrated culture supernatant and analyzed in flow cytometry for an intact (IgM Fc^+^ F(ab')_2_^+^) or cleaved (IgM Fc^+^ F(ab')_2_^−^) IgM BCR (gating described in **Figure 1A**). A significant reduction of IgM Fc^+^ F(ab')_2_^+^ B cells was detectable after treatment with *S. suis* wt supernatant for 45 min and increased to approximately thirty percent when incubated for 4 h (**Figure 1C**). In contrast, there was no detectable effect on IgM Fc^+^ F(ab')_2_^+^ B cells after incubation with concentrated *S. suis* 10Δide*_Ssuis_* ∇ide*_Ssuis_* _C195S culture supernatant. These results demonstrate that not only rIde*_Ssuis_* _homologue (as shown above) but also Ide*_Ssuis_* expressed by *S. suis* is able to cleave the porcine IgM BCR.

To verify that the measured IgM signals originate from the IgM BCR and not secreted and then surface-bound soluble IgM, i.e. bound to FcµR (18, 33, 34), we investigated B cell culture supernatants in anti-IgM Western blot analysis. Cells were treated with rIde*_Ssuis_* _homologue or rIde*_Ssuis_* _homologue_C195S, followed by removal of the recombinant proteins and cleavage products and incubation for 21 h. Cell culture supernatant was collected directly, 1 h and 21 h after washing and investigated for soluble IgM. Neither cells preincubated with rIde*_Ssuis_* _homologue nor rIde*_Ssuis_* _homologue_C195S showed IgM-specific protein bands at any time point (**Supplementary Figure 6**). These findings indicate that the cells do not produce soluble IgM that could bind to the cell surface. This is in accordance with the interpretation that the detected IgM F(ab')_2_ signals rely on the expression of the IgM BCR and not on receptor-bound secreted IgM. Additionally, we investigated the capacity of PBMCs to bind soluble IgM to further exclude that soluble and subsequently surface-bound IgM was contributing to the IgM signals measured by flow cytometry. To this end, porcine PBMC were incubated with 0.5 mg/mL soluble porcine IgM and analyzed for IgM Fc^+^ cells by flow cytometry. Cells positive for myeloid marker CD172a, known to express IgM receptors on the cell surface (18), were used as positive control. No significant effect on the percentage of IgM Fc^+^ porcine B cells (IgM Fc^+^ CD79α^+^) could be measured after IgM preincubation (**Supplementary Figure 7**). In contrast, CD172a^+^ cells showed a significant increase in the percentage of IgM Fc^+^ cells as well as in the median fluorescence intensity of the IgM Fc signal, indicating that porcine CD172a^+^ cells efficiently bind soluble IgM on the cell surface in contrast to B cells. This is also consistent with our interpretation that the IgM signal of B cells derives from transmembrane IgM as part of the IgM BCR.

In conclusion, these results show for the first time that rIde*_Ssuis_* _homologue cleaves the IgM BCR as present on the surface of B cells (CD79α^+^ CD3^-^) in porcine mandibular lymph node cells and PBMC.

### Complete recovery of IgM B cell receptor expression on the surface of porcine lymph node cells takes approximately 20 hours after rIde*_Ssuis_* _homologue-mediated cleavage

After treatment of human PBMC with IgG-degrading enzyme IdeS of *S. pyogenes*, the number of IgG BCRs takes 16 h to return to pretreatment levels (32). To verify if the porcine IgM BCR is recovered in a similar time course, mandibular lymph node cells were incubated with rIde*_Ssuis_* _homologue or non-functional rIde*_Ssuis_* _homologue_C195S, followed by incubation for up to 20 h. Cells were stained for CD79α, IgM Fc and IgM F(ab')_2_ and analyzed in flow cytometry for intact or cleaved BCR as described above (**Figure 1A**). The number of IgM Fc^+^ F(ab')_2_^+^ B cells in mLN cells increased over time from approximately 20% at 0 h to almost 80% at 20 h after treatment with rIde*_Ssuis_* _homologue. After 20 h, numbers of IgM Fc^+^ F(ab')_2_^+^ cells were comparable between rIde*_Ssuis_* _homologue and rIde*_Ssuis_* _homologue_C195S treated cells (**Figure 2**). These findings demonstrate that the IgM BCR signal takes approximately 20 h to fully recover. The full recovery of the IgM signal on CD79α^+^CD3^-^ B cells after rIde*_Ssuis_* _homologue cleavage further supports the interpretation that this signal is due to the porcine IgM BCR and not to surface-bound soluble IgM.

### Treatment with rIde*_Ssuis_* _homologue specifically abolishes IgM B cell receptor mediated signaling

BCR signaling plays a key role in B cell activation and differentiation. BCR ligand-dependent activation results in the recruitment of protein tyrosine kinases Lyn and Syk and downstream tyrosine phosphorylation of phospholipase C-γ2 (PLC-γ2) (35).

**Figure 3:**
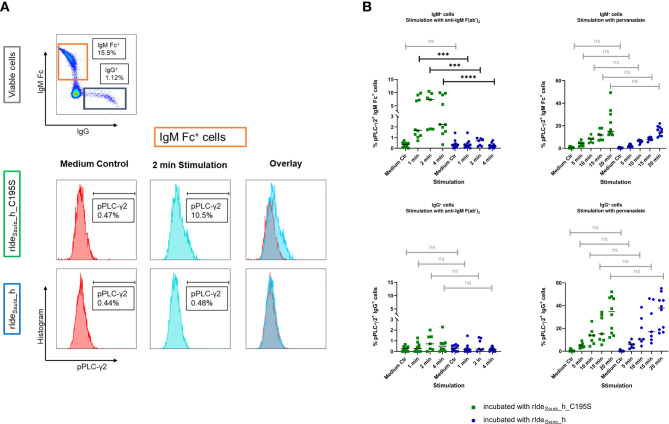
Treatment with rIde*_Ssuis_* _homologue abolishes IgM B cell receptor mediated signaling after IgM F(ab’)_2_-specific stimulation in IgM^+^, but not IgG^+^ B cells. **(A)** Gating strategy to analyze IgM^+^ and IgG^+^ cells for phosphorylated phospholipase C-γ2 (pPLC-γ2) after treatment with rIde*_Ssuis_* _homologue (rIde*_Ssuis_* _h) or non-functional point-mutated rIde*_Ssuis_* _homologue_C195S (rIde*_Ssuis_* _h_C195S). Representative histograms of IgM Fc^+^ cells are shown. Cells were kept in culture medium as control (lower panels, left hand side) or were stimulated with anti-IgM F(ab’)_2_ (lower panels, middle) and analyzed for the percentage of pPLC-γ2^+^ cells. **(B)** Stimulation with anti-IgM F(ab’)_2_ induces tyrosine phosphorylation of phospholipase C-γ2 (PLC-γ2) in IgM^+^, but not IgG^+^ lymphocytes. “After cleavage of the IgM BCR, there is no longer an increase in pPLC-γ2^+^ IgM^+^ cells detectable. Both cell types can be stimulated with pervanadate, independent of BCR cleavage. Porcine PBMC of 7 to 8-week-old piglets (n = 10) were incubated for 45 min with 4 µg/1x10^6^ cells rIde*_Ssuis_* _homologue (rIde*_Ssuis_* _h) or rIde*_Ssuis_* _homologue_C195S (rIde*_Ssuis_* _h_C195S). After washing, cells were stimulated with anti-IgM F(ab’)_2_ or tyrosine phosphatase inhibitor pervanadate for the indicated times. Cells were stained and analyzed for IgM Fc or IgG and phosphorylated PLC-γ2^+^ by flow cytometry. Same time points were compared with Mann-Whitney test. Bars represent median, significant differences are indicated (p > 0.5 ns, p < 0.001 ***, p < 0.0001 ****).

We hypothesized that IgM BCR cleavage leads to impaired B cell activation. Therefore, we incubated PBMC with rIde*_Ssuis_* _homologue to cleave the BCR or non-functional rIde*_Ssuis_* _homologue_C195S for comparison as described above. Cleavage of the IgM BCR was verified by flow cytometry as described in **Figure 1A**. Next, the cells were treated with different stimuli and analyzed by flow cytometry for tyrosine-phosphorylated PLC-γ2 (pPLC-γ2) as readout parameter for activation of the intracellular downstream signal cascade. IgM BCR specific stimulation was achieved by anti-IgM F(ab')_2_. The gating strategy of IgM^+^ and IgG^+^ cells and representative histograms of IgM^+^ cells after 2 min stimulation with anti-IgM F(ab')_2_ in comparison to medium control are shown in **Figure 3A**. Protein tyrosine phosphatase inhibitor pervanadate intervenes at the level of downstream signal transduction (31) and was used for BCR-independent increase of phosphorylation.

We investigated the stimulatory capacity and the consequence of BCR cleavage by rIde*_Ssuis_* _homologue on the percentage of IgM Fc^+^ pPLC-γ2^+^ cells in PBMC of 7 to 8-week-old piglets and 16-week-old pigs. The cells of the different age groups behaved similarly (**Supplementary Figure 8**). Hence, we thereupon focused on PBMC from 7 to 8-week old piglets as the typical age period for *S. suis* disease.

Stimulated with anti-IgM F(ab')_2_, IgM Fc^+^ cells preincubated with rIde*_Ssuis_* _homologue_C195S showed a significant increase in the percentage of pPLC-γ2^+^ cells in comparison to IgM^+^ cells preincubated with rIde*_Ssuis_* _homologue (**Figure 3B**). These findings demonstrate that IgM BCR cleavage prevents recognition and ligand-dependent activation *via* the F(ab) part and therefore impairs intracellular signaling. As expected, IgG^+^ cells showed no increased phosphorylation of PLC-γ2 after stimulation with anti-IgM F(ab')_2_ as well as no effect of preincubation with functional or non-functional rIde*_Ssuis_* constructs. In contrast, the median fluorescence intensity (MFI) of pPLC-γ2^+^ did not increase after stimulation of IgM^+^ cells preincubated with rIde*_Ssuis_* _homologue_C195S in comparison to IgM^+^ cells preincubated with rIde*_Ssuis_* _homologue (**Supplementary Figure 9**). Taken together, these data indicate that IgM BCR-specific stimulation induces a higher frequency of pPLC-γ2^+^ IgM^+^ cells, while the level of phosphorylation per single cell remains restricted to a certain limit when all PTKs are already recruited.

When treated with pervanadate, both IgM^+^ and IgG^+^ cells showed a time-dependent increase in pPLC-γ2^+^ cells independent of enzyme preincubation (**Figure 3B**). This demonstrates that rIde*_Ssuis_* _homologue cleavage activity only affects the IgM BCR-mediated activation. Following 45 min preincubation with either rIde*_Ssuis_* _homologue or rIde*_Ssuis_* _homologue_C195S, the intracellular downstream signaling cascade appears to be still intact. In contrast to BCR-mediated stimulation, treatment with pervanadate led to a time-dependent increase in frequency as well as in MFI in both IgM^+^ and IgG^+^ cells (**Supplementary Figure 9**). However, IgM^+^ cells do not reach quite the same high levels of phosphorylation as IgG^+^ cells. In conclusion, pervanadate stimulation increased the number of pPLC-γ2^+^ cells as well as phosphorylation levels per cell in the investigated IgM^+^ and IgG^+^ cells with no difference between IgM^+^ cells with intact or cleaved BCR.

Additionally, after stimulation with anti-IgM F(ab')_2_ or pervanadate, cells were gated for IgM^-^ cells and analyzed for the percentage of pPLC-γ2^+^ cells as described above. In addition to IgG^+^ B cells, the IgM^-^ cell population also includes IgA^+^ and IgD^+^ B cells. As expected, IgM^-^ cells showed no increase in pPLC-γ2^+^ cells after IgM-specific stimulation, whereas pPLC-γ2^+^ cells increased significantly after treatment with pervanadate, independent from pretreatment with rIde*_Ssuis_* _homologue or rIde*_Ssuis_* _homologue_C195S (**Supplementary Figure 10**). These results underline the specificity of polyclonal anti-IgM F(ab')_2_ used for stimulation of IgM^+^ B cells *via* the IgM F(ab) part of the BCR.

### rIde*_Ssuis_* _homologue cleaves the IgM BCR and inhibits IgM B cell receptor mediated signaling on both CD21^+^ B2 cells and CD21^-^ cells

**Figure 4:**
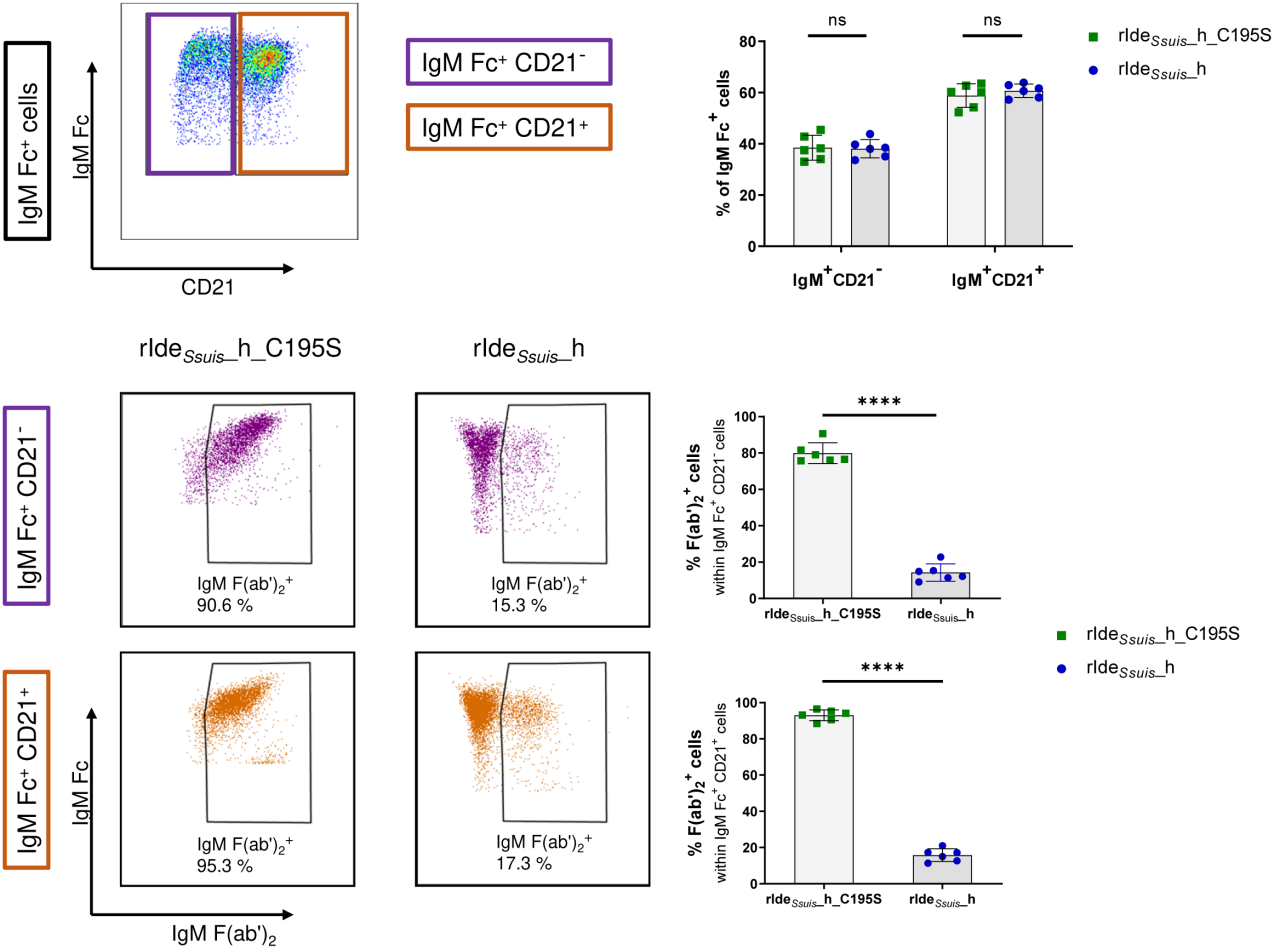
rIde*_Ssuis_* _homologue cleaves the IgM B cell receptor on CD21^+^ B2 cells and CD21^-^ cells. IgM^+^ cells in porcine PBMCs of 8-week-old piglets can be discriminated by CD21 expression (upper panels). Both IgM^+^CD21^-^ B1-like cells (middle panels) and IgM^+^CD21^+^ B2 cells (lower panels) show significant IgM BCR cleavage by rIde*_Ssuis_* _homologue (rIde*_Ssuis_* _h) (4 µg/1x10^6^ cells, 45 min, 37°C) compared to rIde*_Ssuis_* _homologue_C195S (rIde*_Ssuis_* _h_C195S). Pre-gating of IgM Fc^+^ cells was performed as described in **Figure 1A**. Statistical analyses were conducted with unpaired t-test (n = 6, p > 0.05 ns, p < 0.0001 ****).

To investigate Ide*_Ssuis_* cleavage activity on different IgM-expressing B cell subpopulations, i.e. a CD21^+^ B2 population and a CD21^-^ population, containing putative B1-like cells (36), PBMC were treated with rIde*_Ssuis_* _homologue or rIde*_Ssuis_* _homologue_C195S, stained for IgM Fc and CD21 and analyzed in flow cytometry. Both IgM^+^CD21^+^ and IgM^+^CD21^-^ cells showed a significant reduction in IgM F(ab')_2_^+^ cells after incubation with rIde*_Ssuis_* _homologue in contrast to rIde*_Ssuis_* _homologue_C195S treated cells (**Figure 4**). In conclusion, rIde*_Ssuis_* _homologue cleaves the IgM BCR present on B1-like and B2 cell subsets differentiated by CD21 expression.

**Figure 5:**
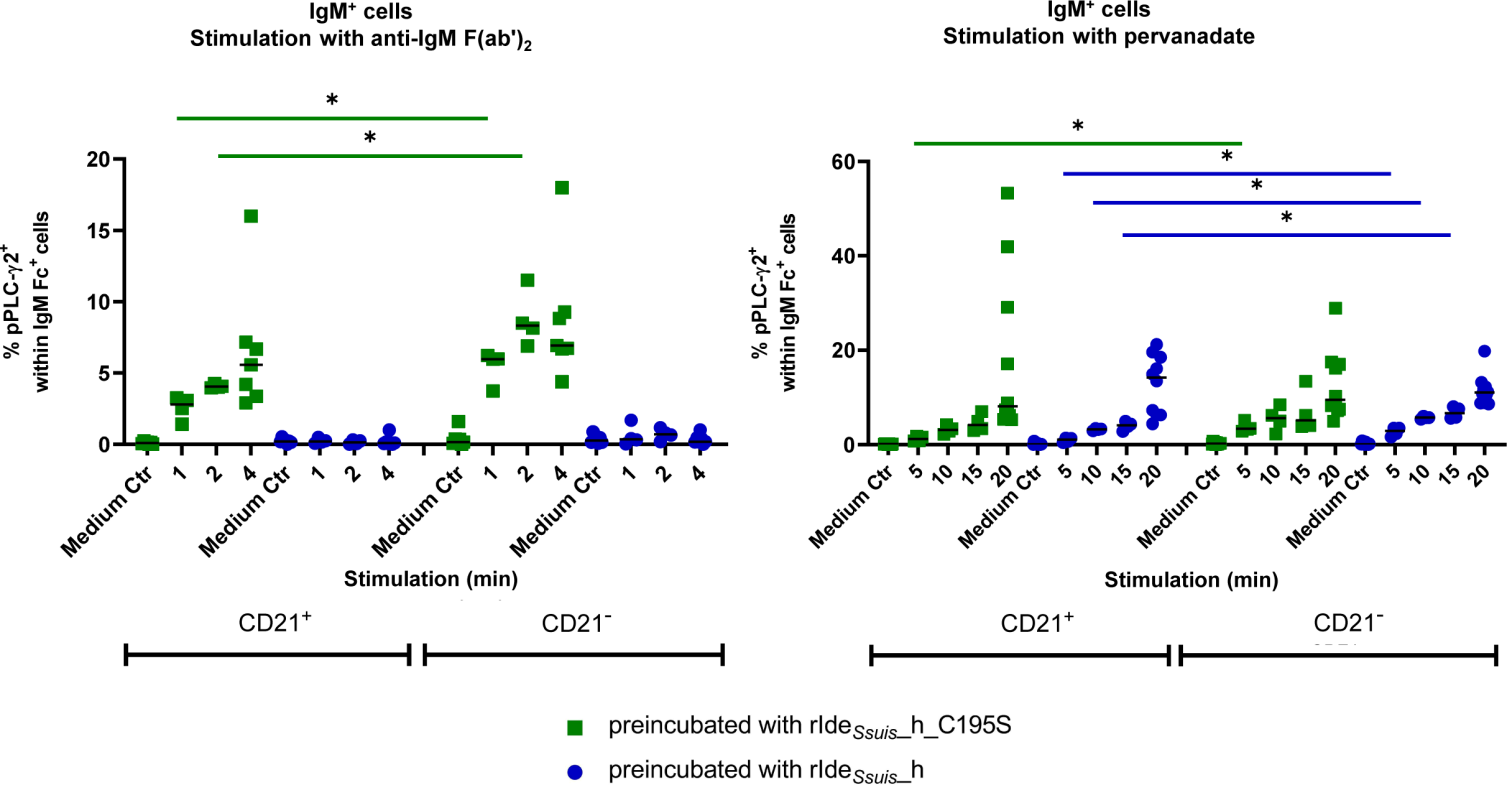
Treatment with rIde*_Ssuis_* _homologue abolishes IgM B cell receptor mediated signaling in CD21^+^ B2 cells and CD21^-^ cells. Porcine PBMC of 7 to 8-week-old piglets (n = 4 - 8) were incubated for 45 min with 4 µg/1x10^6^ cells rIde*_Ssuis_* _homologue (rIde*_Ssuis_* _h) or rIde*_Ssuis_* _homologue_C195S (rIde*_Ssuis_* _h_C195S). After washing, cells were stimulated with anti-IgM F(ab’)_2_ or tyrosine phosphatase inhibitor pervanadate for the indicated times or were kept in cell culture medium without stimulation (Medium Ctr.). Cells were stained and analyzed for IgM Fc, CD21 and phosphorylated PLC-γ2 by flow cytometry. Cells with the same treatment were compared with Mann-Whitney test. Bars represent median, significant differences are indicated (p< 0.1 *). Results of statistical analyses considered not significant are not shown (p > 0.05).

We further assessed the consequence of IgM BCR cleavage on the signaling of CD21^+^ B2 cells compared to CD21^-^ B cells. Cells were pretreated with rIde*_Ssuis_* _homologue or rIde*_Ssuis_* _homologue_C195S before stimulation with specific anti-IgM F(ab')_2_ or protein tyrosine phosphatase inhibitor pervanadate (as described above). Stimulation of both CD21 populations within IgM^+^ cells with anti-IgM F(ab')_2_ after preincubation with rIde*_Ssuis_* _homologue_C195S resulted in a distinct increase in pPLC-γ2^+^ cells in contrast to IgM^+^ cells preincubated with rIde*_Ssuis_* _homologue (**Figure 5**). Significant differences were found comparing CD21^+^ and CD21^-^ cells preincubated with non-functional rIde*_Ssuis_* _homologue_C195S after a stimulation time of 1 min and 2 min, with higher levels of pPLC-γ2^+^cells within CD21^-^ cells. Phosphorylation of PLC-γ2 was completely abolished by preincubation with rIde*_Ssuis_* _homologue, with no significant differences between CD21^+^ and CD21^-^ cells, indicating that both IgM^+^ cell subsets are equally impaired in downstream BCR signaling. After stimulation with pervanadate, both cell types showed an increase in pPLC-γ2^+^ cells independent from preincubation. Statistical analysis found higher percentages of pPLC-γ2^+^ cells within rIde*_Ssuis_* _homologue_C195S-preincubated CD21^-^ cells after a stimulation period of 5 min as well as within rIde*_Ssuis_* _homologue-preincubated CD21^-^ cells after a stimulation period of 5, 10, and 15 min in comparison to CD21^+^ cells. Taken together, significantly more CD21^-^ cells were positive for pPLC-γ2 at specific time points. In summary, these data demonstrate IgM BCR cleavage by rIde*_Ssuis_* _homologue and subsequent impairment of intracellular signaling after ligand-dependent receptor activation on both naïve and innate B cell subsets.

## Discussion

Previous research demonstrated that the cysteine protease Ide*_Ssuis_* specifically cleaves soluble porcine IgM and reduces complement activation (12, 16, 17). Whether Ide*_Ssuis_* is also able to cleave transmembrane IgM within the BCR complex has not been investigated so far. We were able to show that the IgM BCR is efficiently cleaved by recombinant functional Ide*_Ssuis_* derivatives as well as by Ide*_Ssuis_* secreted in culture supernatant (**Figure 1**).

IgM BCR recovery on the cell surface after cleavage by rIde*_Ssuis_* _homologue was investigated over a time period of 21 h. To ensure returning IgM F(ab')_2_ signals derive from transmembrane IgM within the BCR complex and not secreted and subsequently surface-bound IgM, we investigated soluble IgM in cell culture supernatants after treatment with rIde*_Ssuis_* _homologue. Even after an incubation time of 21 h, there was still no soluble IgM detectable, which could potentially bind to the B cell surface *via* IgM Fc receptors and account for the return of measured IgM F(ab')_2_ signals (**Supplementary Figure 6**). This finding is consistent with the study of Braun et al. reporting IgM secretion into culture supernatant only after stimulation, but not spontaneously (36). In addition, we found no increased IgM signal on the surface of porcine B cells after incubation with purified soluble IgM (**Supplementary Figure 7**). Taken together, our data indicate recovery through expression of new BCRs rather than secretion of soluble IgM and recruitment to the cell surface by Fc receptors (18). Interestingly, after rIde*_Ssuis_* _homologue cleavage, recovery of the IgM BCR on the cell surface takes at least 20 h in mandibular lymph node cells (**Figure 2**). These findings record similarities to the recovery period of B cells after treatment with IdeS of *Streptococcus pyogenes*, with studies recording that IgG F(ab)^+^ peripheral blood B cells take 16 h to return to pretreatment levels (32). In comparison, recovery of the IgM BCR takes even longer. The comparatively long recovery period suggests potential biological effects in terms of interfering with the hosts immune response.

IgM BCR cleavage by rIde*_Ssuis_* _homologue prevents downstream signaling after F(ab')_2_-specific receptor stimulation, thus impairing IgM B cell activation following antigen binding (**Figure 3B**). Similarly, treatment of human B cell lymphoma cells with IdeS inhibits IgG BCR signaling after stimulation *via* the F(ab) part (32). As IgM is the main BCR on B1 cells and naïve conventional B2 cells (37), IgM BCR cleavage could interfere with the innate immune response as well as the first rapid IgM response in the context of adaptive immunity. On the other hand, the intracellular pathways remain uninfluenced by BCR cleavage. This could be shown by using the tyrosine phosphatase inhibitor pervanadate, where no differences in phosphorylation of PLC-γ2 between B cells with rIde*_Ssuis_* _homologue-mediated BCR cleavage and treatment with rIde*_Ssuis_* _homologue_C195S were found. Pervanadate intracellularly inhibits PTPs, thereby shifting the balance towards activating tyrosine kinases (29). This demonstrates that Ide*_Ssuis_* is able to block B cell activation specifically *via* the BCR.

Mature naïve B cells exit the bone marrow and migrate to secondary lymphoid organs like lymph nodes where they encounter antigen (38, 39). Antigen binding to the BCR of naïve B cells initiates intracellular downstream signaling, antigen internalization and presenting of processed antigen on MHC II molecules (37). As BCR signals and recognition of the antigen-MHC II complex by specific T helper cells lead to proliferation, class switch and differentiation into antibody-secreting or memory B cells, we speculate that infection of lymph nodes with *S. suis* might lead to impaired B cell differentiation if enough Ide*_Ssuis_* is expressed to efficiently cleave the IgM BCR. As encapsulated *S. suis* is also known to modulate MHC-II restricted antigen presentation and cytokine secretion of dendritic cells, leading to an impaired CD4^+^ T cell activation (40, 41), this important invasive pathogen might substantially interfere with the adaptive immune response of its porcine host. We speculate that this interference with the adaptive immune response contributes to survival of an invasive *S. suis* strain in the tonsils of convalescent pigs (42).

In B cell development, antigen-independent BCR signaling is important for maintaining tonic signals, which are required for B cell differentiation, maturation and survival at different key checkpoints during development (29, 35). The permanent loss of BCRs on peripheral immature and mature B cells results in cell death (43). It is conceivable that persistent infection of lymph nodes with *S. suis*, expressing high enough Ide*_Ssuis_* levels to efficiently cleave the IgM BCR, leads to an impaired germinal center response. This might interfere with the development into long-living plasma cells, memory cells or even result in B cell anergy or death.

Karlsson et al. compared samples from human patients locally or systemically infected with *Streptococcus pyogenes* to healthy controls and found a specific change in the proteome of the tonsillitis patients (44). The tonsillitis swaps contained much higher levels of IdeS-specific IgG cleavage products compared to plasma from sepsis patients, indicating higher IdeS cleavage efficacy in local infection sites with low IgG levels. Similarly, mucosal surfaces in humans (45) and pigs are characterized by high IgA, but only low IgM levels in comparison to the blood (46). As mucosal surfaces of the upper respiratory tract are the natural environment for *S. suis* and represent an IgM-low microenvironment, we hypothesize an advantage in colonization and survival for *S. suis* by efficient IgM cleavage by Ide*_Ssuis_* on those surfaces.

*S. pyogenes* is commonly found in the human pharynx, residing as a commensal or causing local infections. Similarly, *S. suis* frequently colonizes the upper respiratory tract of pigs. Madsen et al. described a model for aerogenous infection, in which pigs were exposed to aerosolized acetic acid followed by aerosolized *S. suis* (47). After challenge with *S. suis* serotype 2, bacterial antigen was detectable in the crypts and subepithelial tissue of the tonsils (48). This location is compatible with bacterial colonization and subsequent invasion across the epithelium. Some infected pigs developed clinical signs, with *S. suis* detectable also in the tracheobronchial lymph node, the mandibular lymph node and other inner organs. These findings led the authors to assume invasion *via* the tonsils and lymphogenous spread to the regional lymph nodes, but invasion *via* the lower respiratory tract could not be ruled out completely (49). However, regional lymph nodes seem to be a key point in the process of colonization progressing to invasive disease. As for *S. suis*, draining lymph nodes of colonized mucosal surfaces, like mandibular or upper cervical lymph nodes, are important sites of encounter with the hosts immune system. Accordingly, we studied the cleavage of the IgM BCR on lymph node cells which are part of the early interaction with the pathogen during local infection and on PBMC which get involved later in infection during bacteremia. Palatine tonsils are an important reservoir for invasive *S. suis* strains and are discussed as a potential route of entry (49, 50). B cells are naturally present in both tonsils and lymph nodes, so modification of the IgM BCR signaling by secreted Ide*_Ssuis_* would not only be conceivable but also highly biologically relevant.

Of note, this study demonstrates IgM BCR cleavage on different B cell populations *in vitro* but it remains to be shown that the IgM BCR is cleaved during infection *in vivo* and that this is of biological relevance. As *S. suis* is known to bind efficiently to different porcine immune cells present in porcine blood (51), we consider it likely that IgM BCR cleavage occurs *in vivo*.

The porcine IgM^+^CD21^+^ B cell subset is described to consist mostly of naïve B2 cells, whereas IgM^+^CD21^-^ B cells represent innate immune cells with B1-like characteristics (36). In this study, we found antigen-mediated BCR signaling in IgM^+^CD21^+^ and IgM^+^CD21^-^ cells to be equally impaired by rIde*_Ssuis_* _homologue receptor cleavage (**Figure 5**).

B1 cells produce IgM as natural antibodies independent from infection and are accountable for most of the circulating IgM, but can also actively respond to T cell-independent antigenic stimulation, especially to encapsulated bacteria (37). B1 cells are mobilized in response to pathogens and migrate to secondary lymphoid organs like lymph nodes, where they secrete IgM antibodies (52). Thus, B1 cells represent the first line of defense in bacterial infection and link innate and adaptive immunity. This underlines the potential important role of IgM in *S. suis* pathogenesis and the putative advantage of Ide*_Ssuis_* expression for colonization, when *S. suis* encounters the host`s innate immune response.

Additionally, Ide*_Ssuis_* has been demonstrated to be a protective antigen against mortality induced by experimental infection with a *S. suis cps2* or a *cps9* strain (53, 54). There might be different reasons for the high protective efficacy. As shown by Seele et al., vaccination with rIde*_Ssuis_* induces antibodies neutralizing the IgM protease activity (53), which might reduce complement evasion and modification of B cell function as indicated in this paper.

Pathogens have developed a multitude of mechanisms to interfere with B cell functions like presentation of antigens, cell survival or polyclonal B cell activation. Typical examples are Epstein-Barr virus, cytomegalovirus, smallpox virus or *Brucella*, but also parasites like *Trypanosoma* ssp. or *Plasmodium falciparum* (52, 55–57). The modulation of B cells by interfering with BCR signaling seems to be a less common strategy of pathogens. Epstein-Barr Virus induces tyrosine phosphorylation and activation of PLC-γ2 by expression of the latent membrane protein 2A (LMP2A) and therefore mimics BCR signaling events (58). *Yersinia pseudotuberculosis* expresses tyrosine phosphatase YopH, which blocks the tyrosine phosphorylation cascade in early BCR signaling, leaving B cells unable to upregulate surface costimulatory molecule B7.2 in response to antigenic stimulation (59). However, cleavage of the BCR is only described for the IgG protease IdeS of *Streptococcus pyogenes* (32). This current study demonstrates that the IgM protease Ide*_Ssuis_* of *S. suis* is able to cleave the IgM BCR, resulting in impaired IgM B cell signaling after receptor stimulation. We are not aware of any other host-pathogen interaction directly targeting the IgM BCR despite its crucial role in B cell function. The Ide*_Ssuis_* gene is highly conserved in *S. suis* (https://blast.ncbi.nlm.nih.gov/Blast.cgi) and Ide*_Ssuis_* is expressed by *S. suis* of different serotypes and sequence types (12). Therefore, we postulate that Ide*_Ssuis_* plays an important role in persistent local *S. suis* infection and colonization by modulating the pig’s B cell dependent immune response. However, this hypothesis still needs further *in vivo* investigations.

## Data availability statement

The raw data supporting the conclusions of this article will be made available by the authors, without undue reservation.

## Ethics statement

All animal experiments were conducted by veterinarians and in accordance with the principles outlined in the European Convention for the Protection of Vertebrate Animals Used for Experimental and other Scientific Purposes and the German Animal Protection Law (Tierschutzgesetz). All animal experiments (collection of blood and lymph nodes) were either approved by the “Landesdirektion Sachsen” (permit number A09/19 and 25-5131/490/5) or the “Niedersächsisches Landesamt für Verbraucherschutz und Lebensmittelsicherheit” (permit number 25-5131/470/13), which includes review through the registered ethics committees for animal experiments of the two institutions.

## Author contributions

Supervision, resources, project administration: CB, GA, UM. Methodology, Investigation, Software, Data analysis: AB, NS, UM. Important preliminary experiments: VR. Production of central experimental tools: WS. Visualization: AB. Writing – first draft: AB. Writing – review: CB, GA, NS, UM, VR. All authors contributed to the article and approved the submitted version.
