# Supplementary material for: Immunoglobulin M-degrading enzyme of Streptococcus suis (Ide Ssuis ) impairs porcine B cell signaling
Source: Front Immunol. 2023 Feb 16;14:1122808. doi: 10.3389/fimmu.2023.1122808 (PMC9980343; doi:10.3389/fimmu.2023.1122808)
Supplement: Supplementary file 1 [file DataSheet_1.pdf]

Supplementary Material

Immunoglobulin M-degrading enzyme of *Streptococcus suis* (Ide_{Ssuis}) impairs porcine B cell signaling

A. K. Breitfelder[1], W. Schrödl[1], V. Rungelrath[1], C. G. Baums[1], G. Alber[2], N. Schütze[2], U. Müller[2]*

*** Correspondence:** uwe.mueller@bbz.uni-leipzig.de

1 Supplementary Tables and Figures

Supplementary Table 1 Antibodies and antibody dilutions used in Western blot analysis.

Supplementary Table 2 Antibodies used in flow cytometry.

Supplementary Table 3 Agents used for B cell stimulation.

Supplementary Figure 1 Schematic depiction of recombinant Ide_{Ssuis} variants.

Supplementary Figure 2 Ide_{Ssuis} cleaves the IgM BCR on PBMCs and mandibular lymph node cells in piglets. (A) mandibular lymph node cells (B) PBMC.

Supplementary Figure 3 The IgM Fc part is not affected by treatment with $rIde_{Ssuis}$ or *S. suis* culture supernatant. (A) recombinant proteins (B) culture supernatants.

Supplementary Figure 4 Western blot analysis of IgM cleavage activity in culture supernatants.

Supplementary Figure 5 Western blot analysis of culture supernatant Ide_{Ssuis} and recombinant proteins.

Supplementary Figure 6 IgM is not detectable in B cell culture supernatants after incubation with $rIde_{Ssuis}$_homologue. (A) mandibular lymph node cells (B) PBMC.

Supplementary Figure 7 Purified soluble IgM binds to porcine myeloid cells but not to porcine B cells.

Supplementary Figure 8 Comparison of stimulation after BCR cleavage in piglets and pigs.

Supplementary Figure 9 Stimulation after BCR cleavage in IgM^+ and IgG^+ cells.

Supplementary Figure 10 Stimulation of IgM^- cells after BCR cleavage.

1.1 Supplementary Table 1: Antibodies and antibody dilutions used in Western blot analysis

Detection of	primary antibody				secondary antibody			
	specificity	source	conjugation	dilution	specificity	source	conjugation	dilution
porcine IgM	anti-pig IgM (Bethyl Laboratories, cataloge A100-117P)	goat[1]	POD[2]	1:4000				
porcine IgM	anti-pig IgM heavy chain (Novus, cataloge NBP2-42699H)	goat[1]	POD[2]	1:4000				
Ide_{Ssuis}	$rIde_{Ssuis}$	rabbit[1]	-	1:1000	anti-rabbit IgG (Dianova, cataloge 111-035-008)	goat	POD[2]	1:50,000
Ide_{Ssuis}	wt Ide_{Ssuis}	rabbit[1]	-	1:1000	anti-rabbit IgG (Dianova, cataloge 111-035-008)	goat	POD[2]	1:50,000

[1]polyclonal antibody [2]peroxidase

1.2 Supplementary Table 2: Antibodies used in flow cytometry

Name and clone	Antigen	Source and catalog	Conjugation	Concentration/ $4x10^5$ cells
Mouse anti-pig IgM<br>Clone K521C3	IgM Fc	Bio-rad<br># MCA637GA	pure	0.1 – 0.15 µl
Purified mouse IgG1 Isotype Ctr<br>Clone MG1-45	Isotype Ctr IgM Fc	Biolegend<br># 401402	pure	0.1 – 0.15 µl
anti-mouse IgG1<br>clone RMG1-1	Mouse IgG1<br>(secondary Ab for IgM Fc)	Biolegend<br># 406616	Brilliant Violet 421™	0.25 µl
mouse anti-pig<br>Monocyte/Granulocyte<br>Clone 74-22-15A	CD 172a	BD Pharmingen<br># 561498	FITC	0.5 µl
mouse IgG2b Isotype Ctr<br>Clone MG2b-57	Isotype Ctr CD172a	Biolegend<br># 401206	FITC	0.5 µl
mouse anti-pig CD3ε<br>Clone BB23-8E6-8C8	CD3	BD Pharmingen<br># 561477	PE-Cy™7	0.15 µl
mouse IgG2a Isotype Ctr<br>Clone MOPC-173	Isotype Ctr CD3	Biolegend<br># 400231	PE-Cy™7	0.15 µl
anti-human CD79a<br>Clone HM47	CD 79a	Biolegend<br># 333504	PE	0.5 µl
polyclonal rabbit anti-porcine<br>IgM $F(ab')_2$ preparation	IgM $F(ab')_2$	See Material and Methods	FITC	0.5 µl
donkey anti-rabbit IgG<br>Clone Poly4064	Rabbit IgG<br>(polyclonal),<br>Isotype control for IgM $F(ab')_2$	Biolegend<br># 406403	FITC	0.5 µl

eBioscience™Fixable viability Dye	viability	Invitrogen # 65-0866-14	eFluor™506	100 µl 1:500 diluted
mouse anti-PLC-γ2 Clone K86-689-37	Phospholipase C-γ2	BD Biosciences # 558498	Alexa Fluor® 647	2 µl
mouse IgG1 isotype control Clone MOPC-21	Isotype control PLC-γ2	BD Biosciences # 557882	Alexa Fluor® 700	2 µl
Anti-porcine IgG msAB (MT424)	Porcine IgG	Mabtech # 3151-6-250	Biotin	0.3 µl
Streptavidin	Secondary ab for porcine IgG	Biolegend # 405229	Brilliant Violet 605™	0.1 µl
mouse IgG2a isotype ctr Clone MOPC-173	Isotype control for porcine IgG	Biolegend # 400240	Biotin	0.3 µl
CD21 antibody, anti-human Clone REA940	CD21	Miltenyi Biotech # 130-115-514	Biotin	0.5 µl
Streptavidin	Secondary Ab for CD21	Miltenyi Biotech # 405203	PE	0.1 µl

1.3 Supplementary Table 3: Agents used for B cell stimulation

Name	Target	Conjugation	Concentration/4x10^5 cells
polyclonal rabbit anti-porcine IgM F(ab')$_2$ preparation	IgM F(ab')$_2$	pure	10 µg
Pervanadate	Protein tyrosine phosphatases	-	0.1 mM

1.1 Supplementary Figure 1

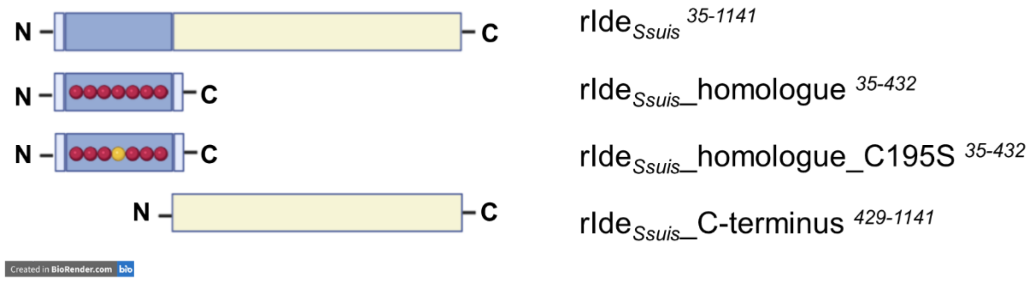

Schematic depiction of recombinant Ide$_{Ssuis}$ variants: the intact IgM protease domain together with the large C-terminus (rIde$_{Ssuis}$), the intact IgM protease domain (rIde$_{Ssuis}$_homologue), the point-mutated, inactive IgM protease domain (rIde$_{Ssuis}$_homologue_C195S) or the C-terminus alone (rIde$_{Ssuis}$_C-terminus). The active IgM protease domain is depicted in dark blue, the C-terminus is depicted in yellow. The amino acids included in the respective variants are superscripted and represented schematically as dots. To construct rIde$_{Ssuis}$_homologue_C195S, cysteine at position 195 was changed to a serine (yellow dot).

1.2 Supplementary Figure 2

A mandibular lymph node cells **B** PBMC

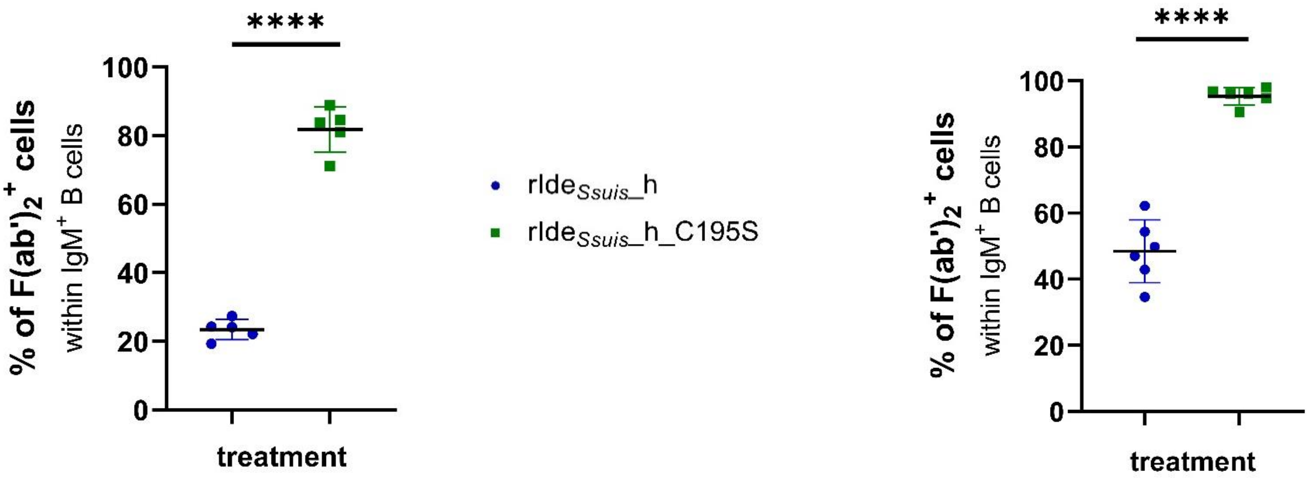

rIde$_{Ssuis}$_homologue cleaves the IgM BCR on PBMCs and mLN cells in piglets.
BCR cleavage with recombinant Ide$_{Ssuis}$_homologue (rIde$_{Ssuis}$_h). Significant reduction of IgM F(ab')$_2^+$ cells by rIde$_{Ssuis}$_h in contrast to rIde$_{Ssuis}$_homologue_C195S (rIde$_{Ssuis}$_h_C195S) (gating as described in Main Figure 1A legend).
mLN cells (**A**, n = 5) or PBMC (**B**, n = 6) of 11-week-old piglets were incubated with 4 µg/1x10^6 cells of rIde$_{Ssuis}$_homologue or rIde$_{Ssuis}$_homologue_C195S for 45 min. Cells were analyzed for the percentage of F(ab')$_2^+$ cells within IgM Fc$^+$ B cells using flow cytometry. Statistical analyses were conducted with unpaired t-test. Bars and error bars represent mean and standard deviation, significant differences are indicated ($p < 0.0001$ ****).

1.3 Supplementary Figure 3

A recombinant proteins

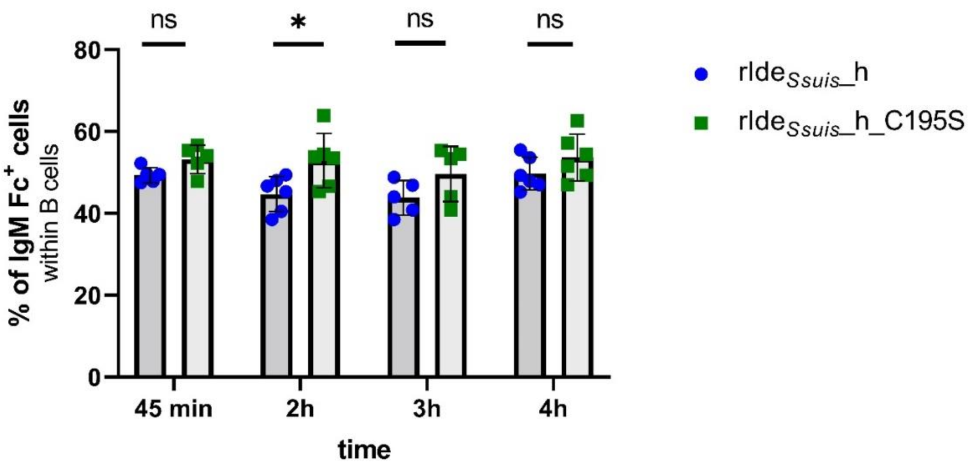

B culture supernatants

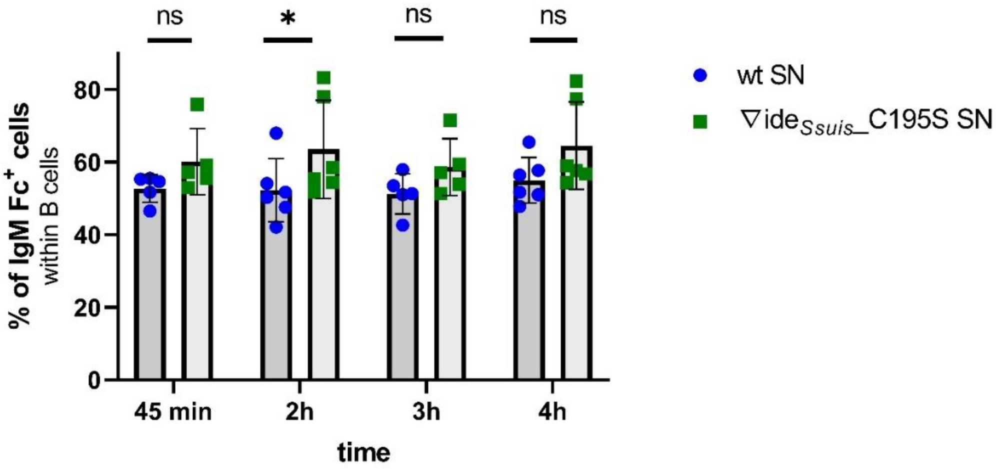

The IgM Fc part is not affected by treatment with rIde$_{Ssuis}$ or *S. suis* culture supernatant.
Cells from porcine mandibular lymph nodes of 11-month-old pigs (n = 6) were incubated with (**A**) rIde$_{Ssuis}$_homologue (rIde$_{Ssuis}$_h), rIde$_{Ssuis}$_homologue_C195S (rIde$_{Ssuis}$_h_C195S) or (**B**) 24-fold concentrated culture supernatant (SN) of *S. suis* 10 (wt) or *S. suis* 10Δide$_{Ssuis}$∇ide$_{Ssuis}$_C195S (∇ide$_{Ssuis}$_C195S) for 45 min, 2h, 3h or 4h. Cells were analyzed for the percentage of IgM Fc^{+} cells within B cells using flow cytometry.
Statistical analyses were conducted with (**A**) unpaired t-test or (**B**) Mann-Whitney test. Bars and error bars represent mean and standard deviation, significant differences are indicated (p >0.05 ns, p < 0.05 *).

1.4 Supplementary Figure 4

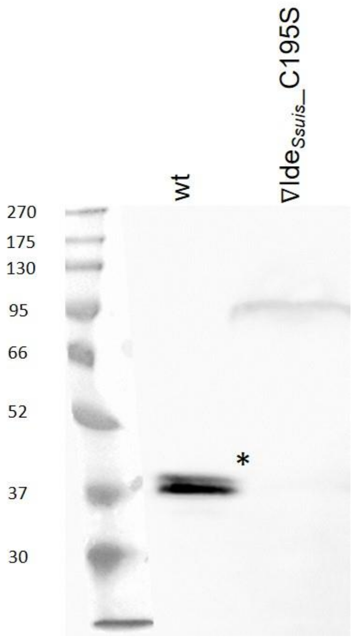

Western blot analysis of IgM cleavage activity in culture supernatants.
Culture supernatants of *S. suis* 10 (wt) show IgM cleavage in contrast to culture supernatants of isogenic point mutant *S. suis* 10 ΔIde$_{Ssuis}$∇Ide$_{Ssuis}$ _C195S (∇Ide$_{Ssuis}$_C195S).
100 µl 24-fold concentrated culture supernatants were incubated with 1:100 prediluted porcine serum and analyzed for cleavage activity in an anti-IgM Western blot. IgM cleavage products are indicated by asterisks. Proteins were separated in SDS-PAGE under reducing conditions with 10 % separating gels. Marker bands in kDa are shown on the left side.

1.5. Supplementary Figure 5

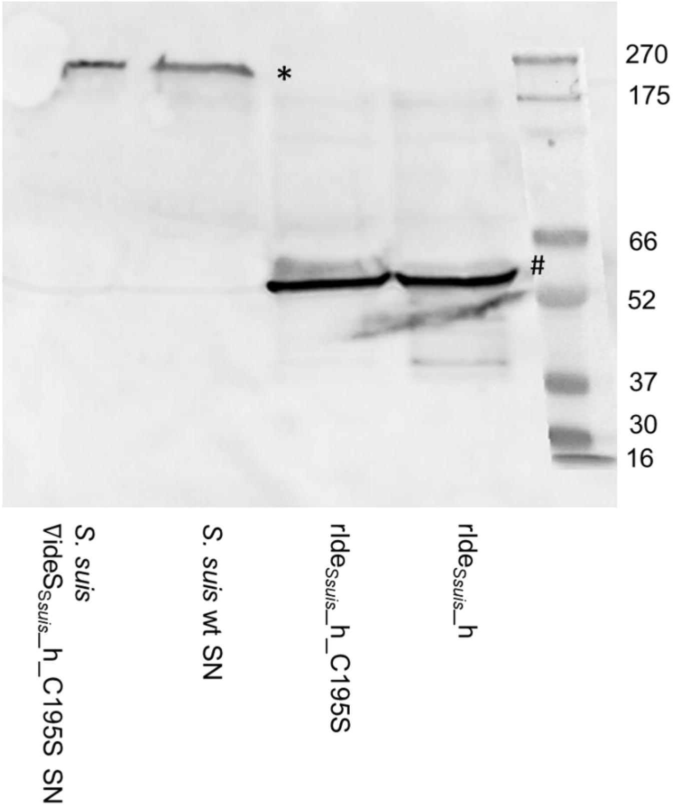

Western blot analysis of culture supernatants and recombinant proteins.
Ide$_{Ssuis}$ of culture supernatants show no signs of degradation after 4h incubation.
Anti-Ide$_{Ssuis}$ Western blot analysis of culture supernatants (SN) of *S. suis* 10 (wt) or *S. suis* 10∇ide$_{Ssuis}$_homologue_C195S (∇ide$_{Ssuis}$_h_C195S) in comparison to recombinant proteins after 4h incubation with mandibular lymph node cells (supernatants from experiment shown in 3A). SDS-PAGE was performed with a 10% polyacrylamide gel under reducing conditions. Marker bands in kDa are shown on the right. Proteins of culture supernatants are indicated by *, recombinant homologue proteins are indicated by #.

1.6. Supplementary Figure 6

A mandibular lymph node cells **B** PBMC

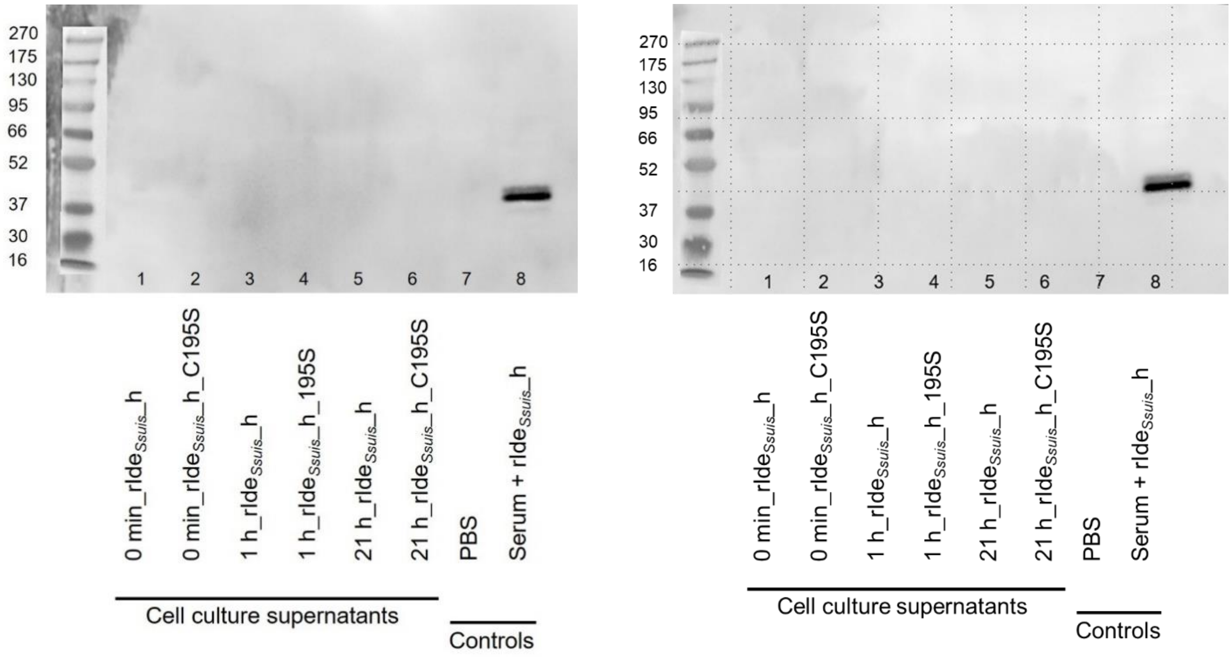

B cells do not secrete IgM after incubation with rIde$_{Ssuis}$_homologue.
Anti-IgM Western blot analysis of culture supernatants of (**A**) mandibular lymph node cells, (**B**) PBMC. Cells were incubated with rIde$_{Ssuis}$_homologue (rIde$_{Ssuis}$_h) or rIde$_{Ssuis}$_homologue_C195S (rIde$_{Ssuis}$_h_C195S). The recombinant proteins and initial cleavage products were removed by washing and the cells resuspended in IMDM medium. Culture supernatant was collected directly, 1 h and 21 h after washing. PBS functioned as negative control, porcine serum incubated with rIde$_{Ssuis}$_homologue as positive control. SDS-PAGE was performed with a 10% polyacrylamide gel under reducing conditions. Marker bands in kDa are shown on the right.

1.7. Supplementary Figure 7

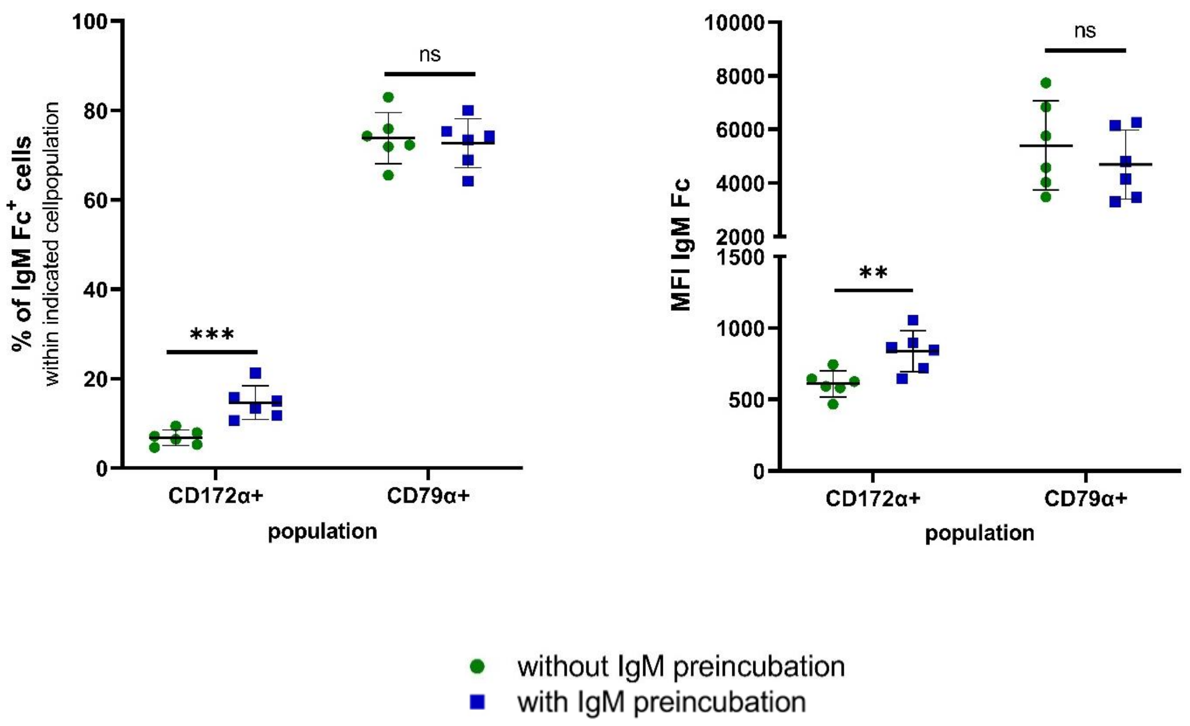

Purified soluble IgM binds to porcine myeloid cells but not to porcine B cells.
PBMC of 6-week-old piglets (n = 6) were incubated with 0.5 mg/ml soluble porcine IgM and analyzed by flow cytometry for the percentage of IgM Fc^+ cells and the median fluorescence intensity (MFI) of IgM Fc within B cells ($CD79\alpha^+$) or myeloid cells ($CD172\alpha^+$).
Statistical analyses were conducted with unpaired t-test. Bars and error bars represent mean and standard deviation, significant differences are indicated ($p > 0.05$ ns, $p < 0.01$ **, $p < 0.001$ ***)

1.8 Supplementary Figure 8

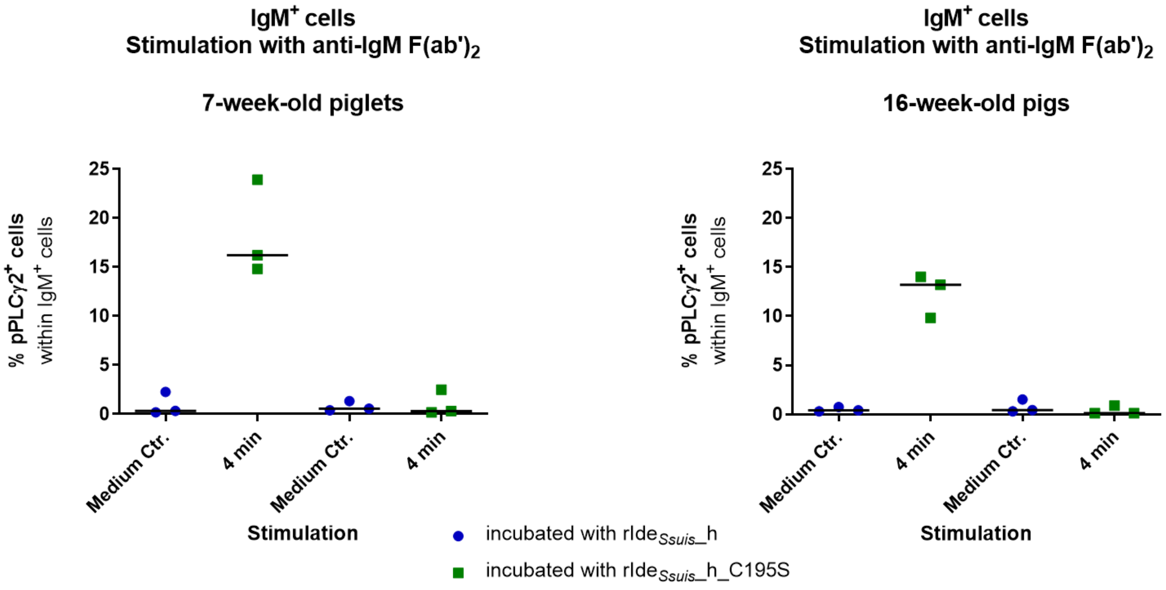

Comparison of stimulation after BCR cleavage in piglets and pigs.
PBMC of 7 to 8-week-old piglets or 16-week-old pigs (n=3) were incubated for 45 min with 4 µg/1x10^6 cells rIde$_{Ssuis}$_homologue (rIde$_{Ssuis}$_h) or rIde$_{Ssuis}$_homologue_C195S (rIde$_{Ssuis}$_h_C195S). After washing, cells were stimulated with anti-IgM F(ab')$_2$ for 4 min and analyzed for phosphorylated PLC-γ2^+ cells by flow cytometry. Treatment with rIde$_{Ssuis}$_homologue abolishes IgM B cell receptor mediated signaling in IgM$^+$ cells in both age groups investigated.

1.9 Supplementary Figure 9

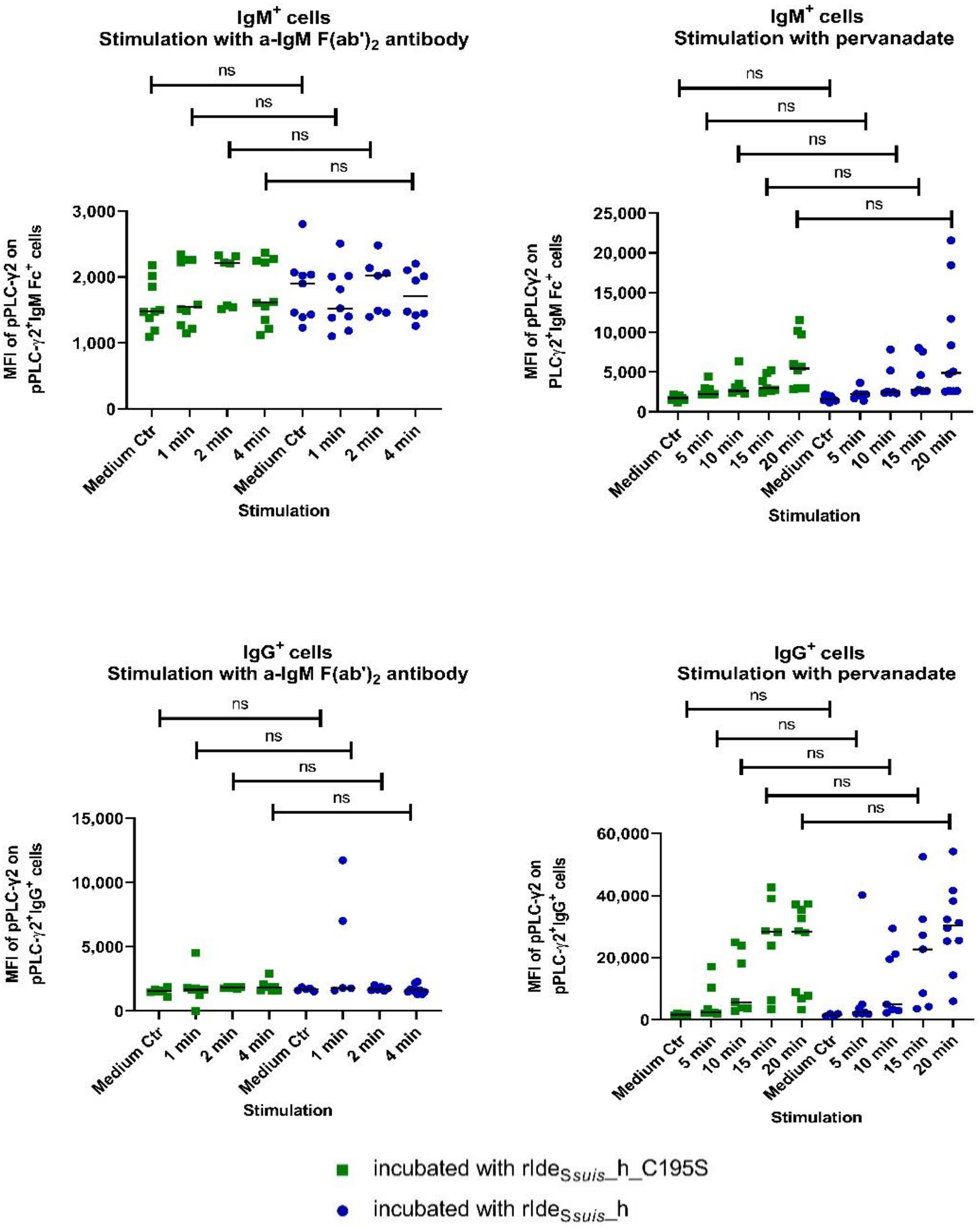

Stimulation after BCR cleavage in IgM+ and IgG+ cells.
Stimulation with anti-IgM F(ab')$_2$ induces no changes in the median fluorescent intensity (MFI) of phosphorylated phospholipase C-γ2 (pPLC-γ2) in IgM$^+$ or IgG$^+$ cells. Stimulation with pervanadate leads to a time-dependent MFI increase. PBMC of 7 to 8-week-old piglets (n = 10) were incubated for 45 min with 4 µg/1x10^6 cells rIde$_{Ssuis}$_homologue (rIde$_{Ssuis}$_h) or rIde$_{Ssuis}$_homologue_C195S (rIde$_{Ssuis}$_h_C195S). After washing, cells were stimulated with anti-IgM F(ab')$_2$ or tyrosine phosphatase inhibitor pervanadate for the indicated times. Cells were stained and analyzed for IgM Fc or IgG and phosphorylated PLC-γ2$^+$ cells by flow cytometry. Cells with same treatment were compared with Mann-Whitney test.
Bars represent median, significant differences are indicated (p > 0.5 ns).

1.10 Supplementary Figure 10

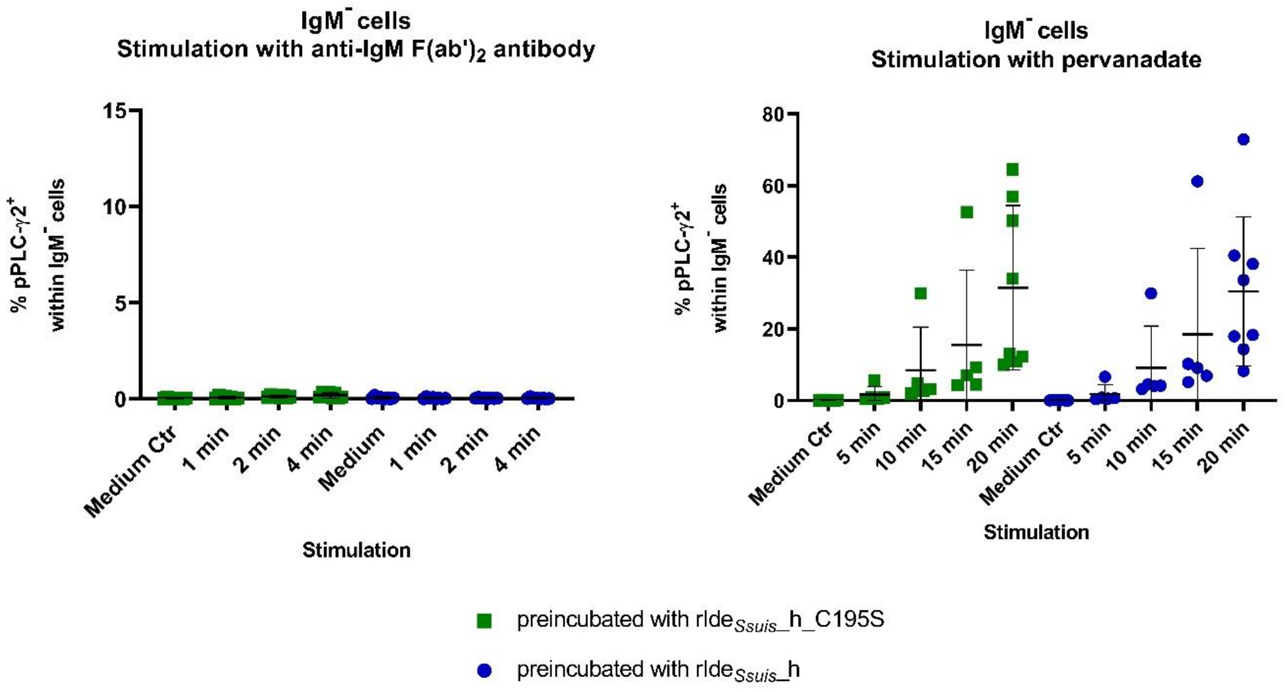

Stimulation with anti-IgM F(ab')2 has no effect in IgM⁻ lymphocytes.
Stimulation with anti-IgM F(ab')2 did not lead to an increase in pPLC-γ2+ cells in IgM⁻ lymphocytes, whereas stimulation with pervanadate led to a time-dependent increase in pPLC-γ2+ cells independent of preincubation with rIde*Ssuis*_homologue (rIde*Ssuis*_h) or rIde*Ssuis*_homologue_C195S (rIde*Ssuis*_h_C195S).
PBMC of 7 to 8-week-old piglets were incubated for 45 min with 4 µg/$1x10^6$ cells rIde*Ssuis*_homologue or rIde*Ssuis*_homologue_C195S. After washing, cells were stimulated with anti-IgM F(ab')2 (n = 8) or tyrosine phosphatase inhibitor pervanadate (n = 5 for 5, 10, 15 min; n = 8 for 20 min) or were incubated with culture medium (n = 8 for medium ctr.) for the indicated times. Cells were stained and analyzed for IgM Fc and phosphorylated PLC-γ2+ cells by flow cytometry.
